# Accurate determination of solvation free energies of neutral organic compounds from first principles

**DOI:** 10.1038/s41467-022-28041-0

**Published:** 2022-01-20

**Authors:** Leonid Pereyaslavets, Ganesh Kamath, Oleg Butin, Alexey Illarionov, Michael Olevanov, Igor Kurnikov, Serzhan Sakipov, Igor Leontyev, Ekaterina Voronina, Tyler Gannon, Grzegorz Nawrocki, Mikhail Darkhovskiy, Ilya Ivahnenko, Alexander Kostikov, Jessica Scaranto, Maria G. Kurnikova, Suvo Banik, Henry Chan, Michael G. Sternberg, Subramanian K. R. S. Sankaranarayanan, Brad Crawford, Jeffrey Potoff, Michael Levitt, Roger D. Kornberg, Boris Fain

**Affiliations:** 1 InterX Inc, 805 Allston Way, Berkeley, CA 94710 USA; 2 Faculty of Physics, Lomonosov Moscow State University, Moscow, 119991 Russia; 3 Department of Chemistry, Carnegie Mellon University, Pittsburgh, PA 15213 USA; 4 Center for Nanoscale Materials, Argonne National Lab, Argonne, IL 60439 USA; 5 Department of Mechanical and Industrial Engineering, University of Illinois, Chicago, IL 60607 USA; 6 Department of Chemical Engineering and Materials Science, Wayne State University, Detroit, MI 48202 USA; 7 Department of Structural Biology, Stanford University School of Medicine, Stanford, CA 94304 USA

**Keywords:** Computational chemistry, Molecular dynamics, Quantum chemistry

## Abstract

The main goal of molecular simulation is to accurately predict experimental observables of molecular systems. Another long-standing goal is to devise models for arbitrary neutral organic molecules with little or no reliance on experimental data. While separately these goals have been met to various degrees, for an arbitrary system of molecules they have not been achieved simultaneously. For biophysical ensembles that exist at room temperature and pressure, and where the entropic contributions are on par with interaction strengths, it is the free energies that are both most important and most difficult to predict. We compute the free energies of solvation for a diverse set of neutral organic compounds using a polarizable force field fitted entirely to ab initio calculations. The mean absolute errors (MAE) of hydration, cyclohexane solvation, and corresponding partition coefficients are 0.2 kcal/mol, 0.3 kcal/mol and 0.22 log units, *i.e*. within chemical accuracy. The model (ARROW FF) is multipolar, polarizable, and its accompanying simulation stack includes nuclear quantum effects (NQE). The simulation tools’ computational efficiency is on a par with current state-of-the-art packages. The construction of a wide-coverage molecular modelling toolset from first principles, together with its excellent predictive ability in the liquid phase is a major advance in biomolecular simulation.

## Abstract (web summary)

Theoretical estimations of solvation free energy by continuum solvation models are generally not accurate. Here the authors report a polarizable force field fitted entirely to first-principles calculations for the estimation of free energy of solvation of arbitrary molecules.

## Introduction

Understanding the energetics of solvation is a fundamental part of describing biophysical processes. The liquid state properties are important in their own right, play a key role in battery design, and are a major part of more structured biological ensembles: e.g., protein shape and behavior, protein–ligand complexes and cell membranes. Because of the overwhelming complexity of ab initio calculations the underlying quantum mechanics must be represented by Newtonian models. The art and science of simulating these systems have been in development since the 1960’s^1,2^ and many force fields that describe proteins and other functional groups have been created and are widely used. However, state-of-the-art wide-coverage molecular force fields^3–9^ in simulation packages that enable free energy computations derive some or all of their parameters by fitting to empirical observables. There are at least two drawbacks to this approach. First, even available experimental data (e.g., densities, heats of vaporization) are insufficient to produce models that describe existing compounds precisely; and there will always be molecules (that, for example, haven’t been synthesized) that will require more precise description than is available from existing inference. Second, if an empirical model’s prediction is erroneous, it is exceedingly difficult to decide exactly which parameter(s) to remove, add, correct or adjust. A major advantage of Quantum Mechanical (QM)-parametrized physics-based molecular models (force fields)^10,11^ is that, with some caveats for molecular size, QM calculations^12^ can be obtained for arbitrary molecules. Another advantage is that prediction errors can be traced to the imprecise description of the interaction energies and rectified in the model. It is therefore highly desirable to create models parameterized entirely from first-principles (ab initio) Quantum Mechanical calculations.

The value of ±0.5 kcal/mol for the desired (“chemical”) accuracy of free energy predictions arises from several considerations. First and foremost, 0.59 kcal/mol is the thermal noise at ambient conditions (room temperature and pressure). This is the inherent fuzziness of our everyday biological world. Additionally, for example, in ligand–protein lead optimization the definition of “incremental lead improvement” is about 0.5 kcal/mol or ~2–3-fold increase in binding affinity.

We have implemented a QM-parametrized force field in a simulation stack that covers arbitrary organic molecules and predicts solvation free energies of molecular systems to accuracy of ~0.3 kcal/mol for neutral species. The predictions in the liquid phase are satisfyingly accurate, and it is also satisfying that the model is created solely from ab initio computational methods without fitting to any experimental data. We demonstrate the predictive ability of the model and simulation machinery by computing solvation free energies for a wide range of chemical functional groups in water and cyclohexane.

## Results

### QM-FF agreement

We start by creating a model that represents the QM energies of the ensemble accurately enough. A description of the intermolecular functional form, the component decomposition, and the parametrization procedure is in Supplementary methods (Quantum mechanical details, force field description, force field functional form of ARROW FF, and parameter fitting), Supplementary Fig. 1 and in references^8,13^. Though models of isolated chemical species with exquisite agreement to QM energies do exist^14,15^, the complexity required by such precision has prevented researchers from describing arbitrary functional groups simultaneously. One of the contributions of this work is determining the degree of faithfulness that is sufficient for modeling the liquid phase of arbitrary organic molecules and mixtures while keeping the model complexity manageable.

The first step is choosing the level and accuracy of the underlying QM computations. We fit the intermolecular interactions to dimer and select multimer QM energies at the highest level of theory practical for large-scale parameterization. This “silver-like standard”^16^ is commonly used as a benchmark in the computational chemistry community, and is within 0.05 kcal/mol from the “gold standard”^16^. More details can be found in Supplementary methods (Quantum Mechanical details).

**Fig. 1:**
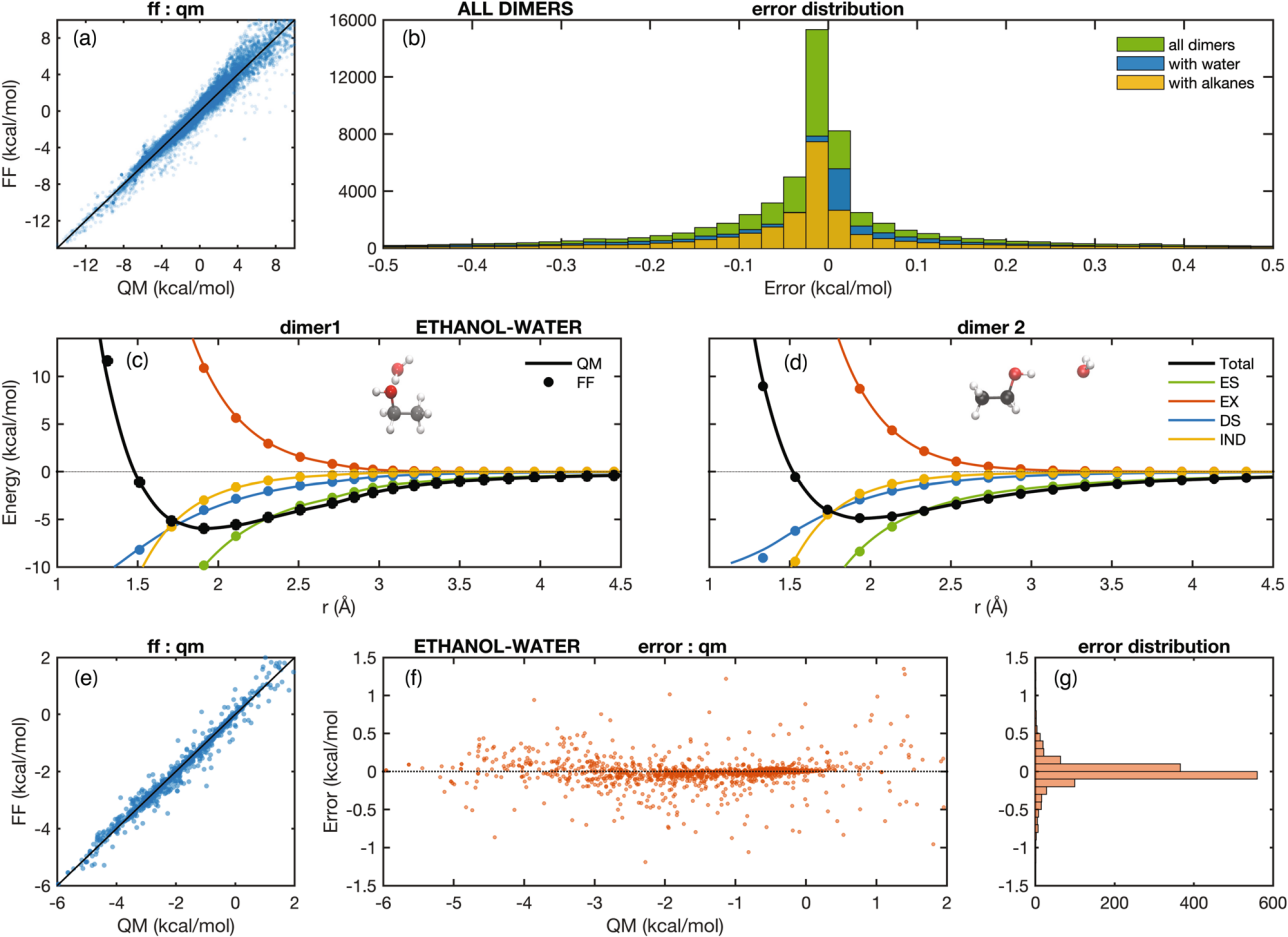
QM: FF energies’ correspondences and deviations. **a** FF vs. QM energies for all the dimers in our training sets. The functional form reproduces the lower energy conformations very well and is designed to permit a larger error in less important high-energy high electron overlap regions. **b** the distribution of errors for our training dimer sets. The MAE of errors are 0.17 kcal/mol for all, 0.19 kcal/mol for dimers with water (total number of dimers = 36,309), and 0.16 kcal/mol for dimers with alkanes (total number of dimers = 25,986): A specific system (ethanol-water) provides a more detailed illustration of model energies and their correspondence with QM. **c**, **d** dissociation curves for primary (**c**) and secondary (**d**) minima of the ethanol-water dimer. QM energies are solid lines and FF values are filled circles. The colors designate the energy components: electrostatics (ES), exchange-repulsion (EX), dispersion (DS) and induction (IND). The agreements for the total energy and for each component are excellent. **e**–**g** Error distributions for the ethanol-water dimer: **e** is analogous to **a**; **f** is a difference plot offering a more detailed view and is projected onto (**g**) the error distribution. The MAE for the errors in this system is 0.08 kcal/mol.

The next step is encapsulating the QM interaction energies in a physics-based analytical model^8,13^. The required faithfulness demands a significant level of complexity from the functional form: polarizability terms enable proper transferability from dimer to bulk energies^17^; multipole descriptions of both the electrostatic^18^ and exchange-repulsion interactions permit a precise fit of the potential energy surface for all dimer orientations^8,19^; a fairly detailed typification accounts for the difference in interaction properties of identical atoms in diverse chemical environments. The force field description including the functional form, and the parametrization workflow and pseudo-code, are discussed in detail in the Supplementary methods. The deviation (MAE) between Quantum mechanical (QM) and force field (FF) energies for all the benchmark dimers and multimers in our training set is 0.17 kcal/mol and the error distribution is centered around zero (Fig. 1a, b, e, f, g). In Fig. 1c, dwe illustrate the QM-FF agreement for a single representative system, a strongly interacting ethanol-water dimer. Additionally, the FF:QM errors for ethanol-water dimers as a function of closest distance are shown in Supplementary Fig. 2. Both the total energies as well as their individual components for this system agree to within 0.1 kcal/mol to their ab initio counterparts. To aid transferability, in addition to reproducing the total energy, we also match the individual components to their corresponding QM counterparts (Fig. 1c, d). To investigate the training-test convergence dependence of dimer space on our force field parameters we conducted this test on a subset of molecules and convergence plots are presented in Supplementary Fig. 4.

Molecular deformations (“bonded interactions”), especially torsions, are critical for correct solvation results because they determine the proper solvent accessibility. A variety of accurate models long established in the field^3,5,6^ as well as brilliant recent developments^20,21^ provide excellent reproduction of the intramolecular energies. We take the functional form of the bonded interactions from MMFF94^3^), with force constants and equilibrium values fitted to QM energies at the MP2/aug-cc-pVTZ level of theory.

### Solvents

We selected water and cyclohexane as our solvents for this benchmark study. Water, of course, is the most ubiquitous molecule in any biophysical model. We chose cyclohexane because it is nonpolar, it equilibrates relatively quickly, and because there is ample reliable experimental data for both cyclohexane (CHEX) solvation free energies, as well as for the cyclohexane/water (CHEX/H_2_O) partition coefficients. Though the two molecules were parameterized with exactly the same procedure as every other functional group, they participate in bulk and thus warrant extra examination of their liquid-state properties.

**Fig. 2:**
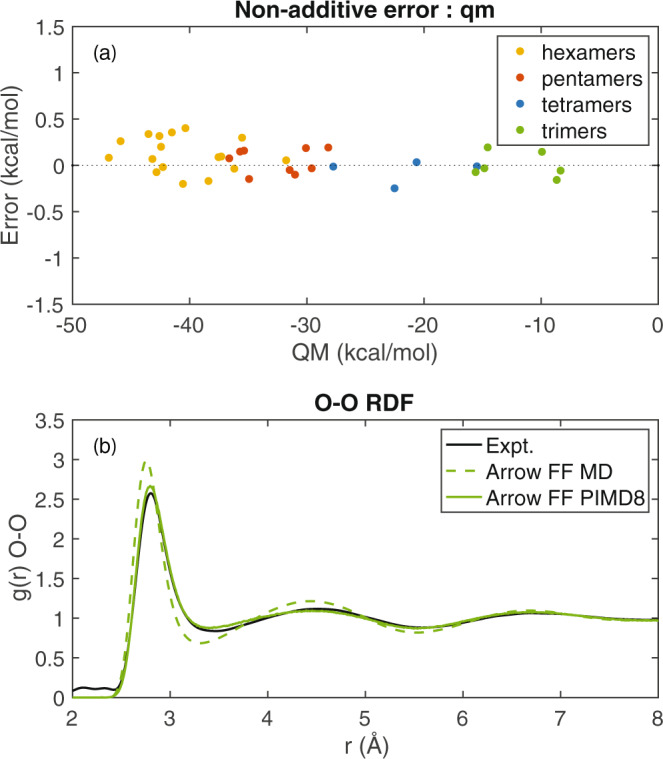
Properties of the ARROW water model. **a** The non-additive many-body error for water multimers vs. their total QM intermolecular energy. All the many-body errors are below 0.5 kcal/mol or 1% of total energy, and below 3% of the many-body contributions. **b** The radial distribution function for the O–O distance in water. The MD RDF (dotted green) is over-structured compared with the experimental curve, and the presence of NQE (solid green) brings the structure of ARROW H_2_O in excellent agreement with the experimental one.

The liquid phase must properly model not only the molecular 2-body interactions described in the previous section, but also the many-body contributions. For water, which is small, polar, and polarizable, the many-body energies are estimated to be a sizable 27% of the total^22^. Figure 2a shows the non-additive energies of select optimized water multimers. Additionally, we also show the non-additive behavior in the case of ethanol–water multimers, see Supplementary Fig. 3. They are in excellent agreement with their reference QM values, confirming that the energy partitioning and the induction terms of our polarizable model capture the non-additive fraction properly.

Biological systems exist mostly at room temperature and pressure, where the shifting interplay between enthalpy and entropy enables the immense variety of biological phenomena. Therefore, it is the free energies of ensembles that are both the most useful and interesting and also the most difficult to predict correctly, and what we focus on here. For solvation, in addition to capturing the enthalpy of interaction with itself and the solute, a solvent model must also reproduce the entropic effects of pushing aside and reordering molecules to create a cavity for placing the solute. This is especially important for water as it is small, highly polar, and, though called a liquid, is highly structured at room temperature and pressure. In Table 1 we list three bulk properties of our solvents: density, heat of vaporization (Hvap) and the highly informative self-solvation. The values for water agree with experimental values to within 3% or better. Additional proof that our model has captured the free energy of cavity creation in water accurately is that the hydration of anthracene, a large, non-polar molecule, is correct to within 2% (0.1 kcal/mol) (Supplementary Data 1a). The derivative of the system Hamiltonian with respect to the alchemical reaction coordinate (<d*H*/dλ) for desolvation of anthracene in water and its accumulated statistical errors are shown in Supplementary Fig. 5. The cyclohexane predictions are slightly less accurate for two reasons: 1) it is a larger molecule so per heavy atoms the energetics are actually very good and 2) we designated its atoms to be the same atom type(s) as linear alkanes (unlike those of smaller, strained, cyclic alkanes), which introduces a slight discrepancy with QM energies. Nonetheless, the bulk energetics of cyclohexane are well within our target accuracy of 0.5 kcal/mol.

**Table 1:** Neat properties and hydration/solvation of water and cyclohexane.

H_2_O	Density (g/cc)	Hvap (kcal/mol)	Hydration (kcal/mol)	Self-solvation (kcal/mol)
expt	0.997	10.51	−6.30	−6.30
ARROW FF (MD)	1.027	11.98	−6.81	−6.81
ARROW FF (PIMD8)	1.027	10.63	−6.13	−6.13

CHEX	Density (g/cc)	Hvap (kcal/mol)	Hydration (kcal/mol)	Self-solvation (kcal/mol)
expt	0.790	7.91	1.20	−4.42
ARROW FF (MD)	0.803	8.04	−0.10	−4.23
ARROW FF (PIMD)	0.786	7.83	1.08	−4.02

Predictions were performed by classical simulations and with inclusion of NQE. All numbers are in good agreement with the experimental values, with PIMD simulations being significantly closer than the classical MD ones. The self-solvation of water is a succinct measure of model accuracy and we recommend its determination for all water models. For water the self-solvation and hydration are obviously identical.

Finally, an excellent measure of how well liquid structure is captured by a model is the radial distribution function (RDF). In Fig. 2b we demonstrate that the ARROW FF reproduces the experimental water oxygen–oxygen (O–O) RDF and, therefore, describes the order of water very well. Additionally, we show that employing eight beads reaches sufficient convergence for the free energies and structural properties (see Supplementary Figs. 7 and 8 and Supplementary Table 4). The agreements for both neat properties (Table 1) and water structure (Fig. 2b) are significantly improved by including NQE^13,15,23^. Satisfyingly, the small errors in initial model parameterization are not amplified through the chain of model construction and simulation machinery.

### Solutes and solvation predictions

**Fig. 3:**
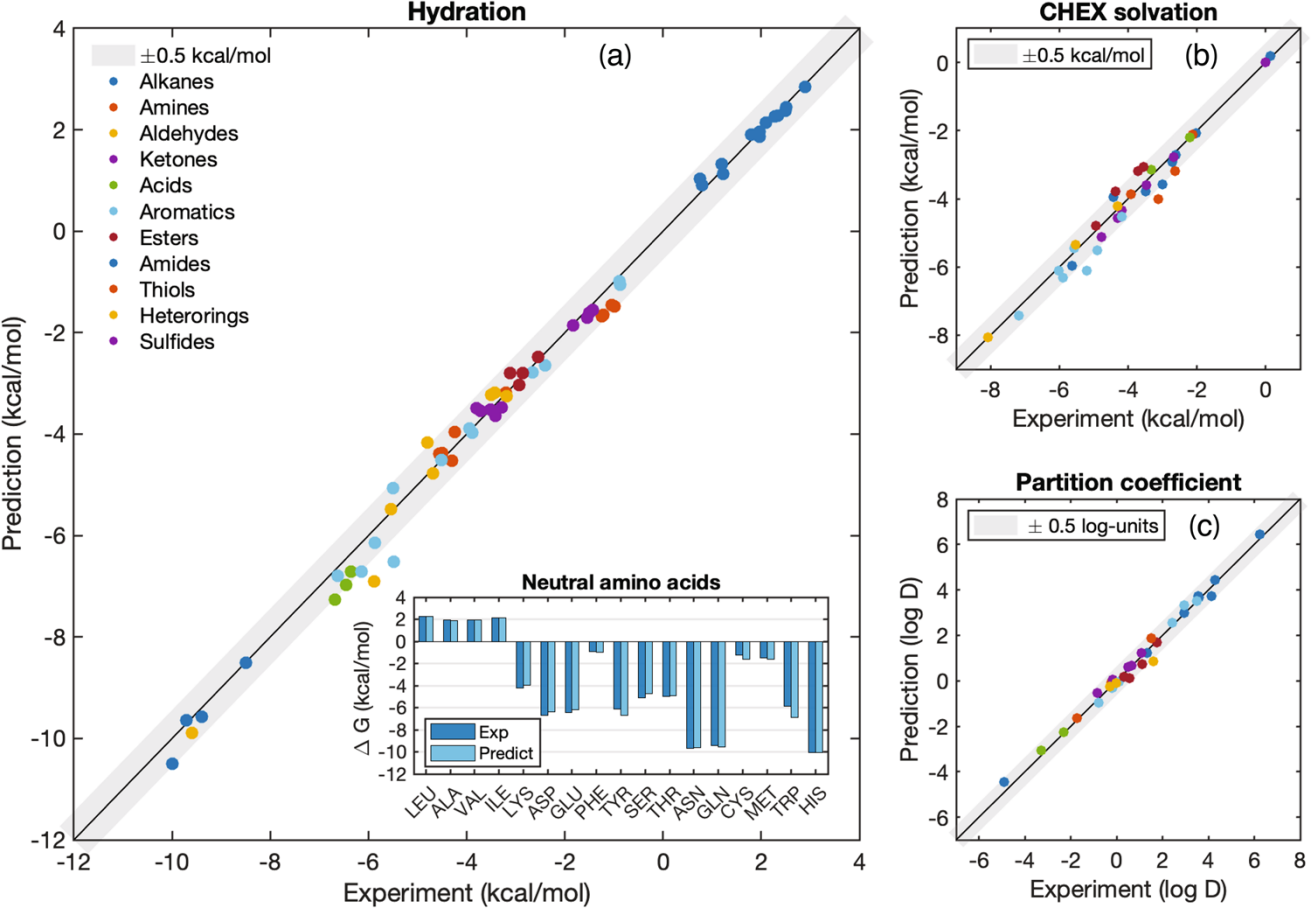
ARROW force field solvation predictions. **a** Predicted vs. experimental free energy of hydration for a diverse set of compounds. The straight line is a line of perfect agreement between experimental and theoretical values, and the gray bar is the range of chemical accuracy. The predicted and experimental free energies of hydration for neutral amino acid analogs are inset. **b** Predicted vs. experimental free energy of solvation in cyclohexane. The molecules here are a subset of those in **a** because only those with experimental values for CHEX solvation can be included. **c** H_2_O/CHEX partition coefficient for the same set as **b**. The free energy predictions are well within chemical accuracy.

We chose representative solutes containing all of the common neutral chemical functional groups: carboxylic acids, alkanes, alkenes, aromatics, aldehydes, ketones, alcohols, amides, esters, thiols, sulfides, disulfides, and heterocycles^24^. The simulations were performed independently by four groups using their own respective computational resources and architectures, and then averaged. The graphical summaries of the solvation and hydration free energies predictions’ are in Fig. 3a, b, and we list the results for each molecule in Supplementary Data 1a. We also provide the free energy results as reproduced by our collaborators in Supplementary Data 1d. Because aqueous protein and protein–ligand systems are of special importance, and because accurate prediction of solvation and desolvation of amino acids is critical for modeling of these systems^25^, we highlight the results for neutral amino-acid analogs separately (Fig. 3a inset), see Supplementary Data 1b for raw data. The partition coefficient is a valuable measure of the model’s simultaneous compatibility with both polar (e.g., aqueous) and non-polar (e.g., membranes and proteins) environments which is crucial for describing bio-molecular systems, and we show it in Fig. 3c.

The proper art of simulation^26,27^ is also essential for obtaining accurate predictions. Accurate treatment of long range electrostatic (e.g., Particle Mesh Ewald^28,29^) and dispersion^27^ interactions, proper thermodynamic modeling (thermostats and barostats)^30,31^, enhanced sampling techniques and the Path Integral formulation of nuclear motion^32–34^ all help to translate the FF-QM agreements to correct free-energy values. Further details are provided in Supplementary methods (Simulation details and protocols). We also provide computational performance of the ARROW FF stack for CPU and CPU+GPU implementations for both classical and path-integral simulations in Supplementary Table 2.

The error (MAE) for the free energies of hydration is 0.2 kcal/mol and for the neutral amino-acid subset is 0.23 kcal/mol. The largest hydration errors seen for o-cresol and 3-methyl-indole are only ~1 kcal/mol. For solvation in cyclohexane the MAE is 0.3 kcal/mol, and for the partition coefficient it is 0.22 log units. These predictions are very good: most are within experimental and simulation uncertainty, and are uniformly correct across a diverse range of chemical groups of varying sizes and interaction strengths.

**Fig. 4:**
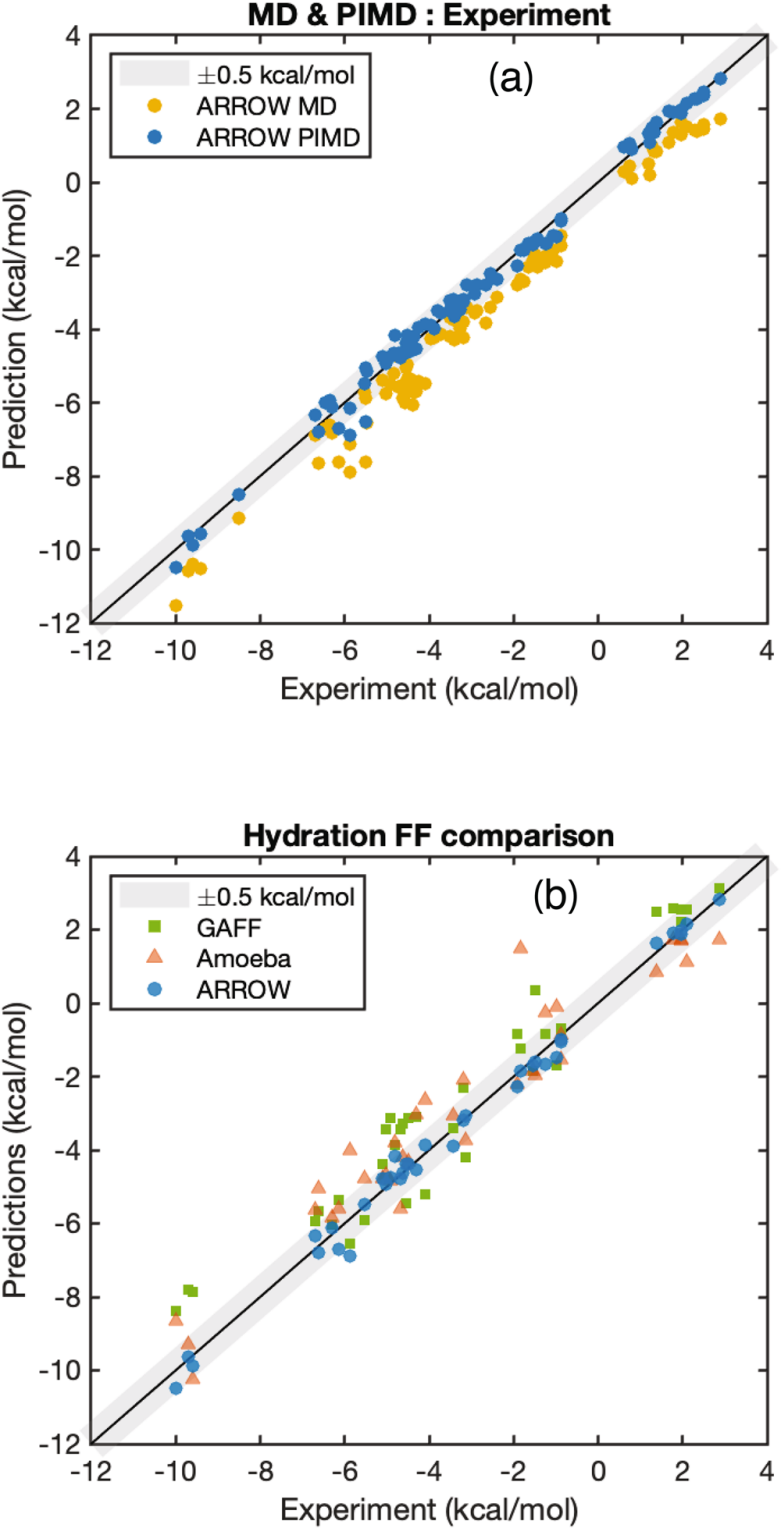
NQE effect and comparison of hydration predictions. **a** A visual comparison of the hydration predictions vs. experimental values for PIMD8 vs. classical MD values. The inclusion of NQE systematically improves the predictions and decreases the overall error (MAE) from 0.78 to 0.2 kcal/mol. **b** A comparison of the hydration free energies to state-of-the art wide coverage Force Fields. The molecules shown include the major functional groups that have been parametrized by all three models and are therefore a subset of those shown in Fig. 3a. The errors (MAE) for GAFF, AMOEBA, and ARROW FF are 0.88, 0.76, and 0.22 kcal/mol, respectively.

We recently highlighted the importance of including NQE when modeling alkanes^13,35^. The results presented in this manuscript suggest that NQE must be taken into account for precision calculations for all molecular systems. We illustrate this in Fig. 4a where we plot the hydration predictions of classical simulations alongside those performed with PIMD. Proper accounting of the quantum nature of nuclear motion systematically shifts the predictions towards the experimental values and improves the prediction error from MAE of 0.78 to 0.2 kcal/mol.

### Comparison with other force fields

The main advance reported in this paper is three-fold: our model is a wide-coverage force field and simulation stack parameterized exclusively from QM data which produces accurate predictions. It is of interest to gauge the relative performance of ARROW FF to existing wide-coverage state-of-the-art models for prediction accuracy. Most of the QM-parameterized FF’s^10,36^ are not currently enabled in a simulation stack which produces free energy predictions, so we selected two widely-used empirical models to compare with. One is GAFF^6^, a representative of the many fixed-charge models, and the other is a polarizable model AMOEBA^9,18^. To avoid reproduction discrepancies the comparison is made on the available published subset of functional groups and is plotted in Fig. 4b. The MAE’s for this subset are, respectively, 0.88 (GAFF AM1-BCC)^37^, 0.76 (AMOEBA)^9^ and 0.22 (ARROW) kcal/mol. A list of molecules and their predicted hydration values for each model is in Supplementary Data 1c. Additionally, in Supplementary Data 1e, Supplementary Table 3, Supplementary Fig. 6, and Supplementary methods (Comparison to Implicit solvent models and Machine learning models) we summarize and discuss the comparative performance of several excellent tools from a variety of methodologies that focus specifically on prediction of solvation energies^38,39^. In Supplementary Data 1f we also show the QM-FF agreement of ARROW FF on the S22 and S66 datasets as well as a comparison with the same for geometry, frequency, non-covalent force field (GFN-FF)^11,39^, the MAE’s for such datasets can be found in Supplementary Table 1.

We have shown that a QM-parametrized, physics-based force field embedded in a simulation and analysis stack predicts the free energies of solvation of arbitrary organic molecules to an accuracy better than thermal noise at room temperature (±0.5 kcal/mol). The correspondence from quantum mechanics to ensemble predictions is established via several important links. First, the benchmark QM calculations need to be of sufficient accuracy. Second, the model should provide a faithful description of the QM potential energy surface (PES), which imposes a significant yet computationally manageable level of complexity on the functional form. Third, the established art of molecular ensemble averaging must be performed with care. Finally, the dynamics of sampling the system should account for nuclear quantum effects. The ARROW FF is likely at the limit of complexity feasible for a wide-coverage analytical force field, and so it is satisfying that this model results in excellent prediction of properties in the liquid phase.

## Supplementary information

Supplementary Information

Description of Additional Supplementary Files

Supplementary Data 1

## Data Availability

The scripts, tools, and data used in this work are available from the corresponding authors and InterX Inc. upon request. The full results’ data has been included in Supplementary Information Tables and further data is also available upon request.

## Code availability

The codes, tools and data needed to reproduce the data presented in this article is available on github https://github.com/freecurve/interx_solvation_suite.

## References

[1] Lifson S, Warshel A (1968). Consistent force field for calculations of conformations, vibrational spectra, and enthalpies of cycloalkane and n‐alkane molecules. J. Chem. Phys..

[2] Levitt M, Lifson S (1969). Refinement of protein conformations using a macromolecular energy minimization procedure. J. Mol. Biol..

[3] Halgren TA (1996). Merck molecular force field. I. Basis, form, scope, parameterization, and performance of MMFF94. J. Comput. Chem..

[4] Jorgensen WL, Maxwell DS, Tirado-Rives J (1996). Development and testing of the OPLS all-atom force field on conformational energetics and properties of organic liquids. J. Am. Chem. Soc..

[5] MacKerell AD (1998). All-atom empirical potential for molecular modeling and dynamics studies of proteins. J. Phys. Chem. B.

[6] Wang J, Wolf RM, Caldwell JW, Kollman PA, Case DA (2004). Development and testing of a general amber force field. J. Comput. Chem..

[7] Mackerell AD (2004). Empirical force fields for biological macromolecules: overview and issues. J. Comput. Chem..

[8] Donchev AG (2008). Assessment of performance of the general purpose polarizable force field QMPFF3 in condensed phase. J. Comput. Chem..

[9] Ponder JW (2010). Current status of the AMOEBA polarizable force field. J. Phys. Chem. B.

[10] Xu P, Guidez EB, Bertoni C, Gordon MS (2018). Perspective: ab initio force field methods derived from quantum mechanics. J. Chem. Phys..

[11] Spicher S, Grimme S (2020). Robust atomistic modeling of materials, organometallic, and biochemical systems. Angew. Chem. Int. Ed. Engl..

[12] Jensen, F. *Introduction to Computational Chemistry* (Wiley, 2017).

[13] Pereyaslavets L (2018). On the importance of accounting for nuclear quantum effects in ab initio calibrated force fields in biological simulations. Proc. Natl. Acad. Sci. USA.

[14] Babin V, Leforestier C, Paesani F (2013). Development of a ‘First Principles’ water potential with flexible monomers: dimer potential energy surface, VRT spectrum, and second virial coefficient. J. Chem. Theory Comput..

[15] Medders GR, Babin V, Paesani F (2014). Development of a ‘First-Principles’ water potential with flexible monomers. III. Liquid phase properties. J. Chem. Theory Comput..

[16] Burns LA, Marshall MS, Sherrill CD (2014). Appointing silver and bronze standards for noncovalent interactions: a comparison of spin-component-scaled (SCS), explicitly correlated (F12), and specialized wavefunction approaches. J. Chem. Phys..

[17] Cieplak P, Dupradeau F-Y, Duan Y, Wang J (2009). Polarization effects in molecular mechanical force fields. J. Phys. Condens. Matter.

[18] Ren P, Ponder JW (2003). Polarizable atomic multipole water model for molecular mechanics simulation. J. Phys. Chem. B.

[19] Van Vleet MJ, Misquitta AJ, Stone AJ, Schmidt JR (2016). Beyond Born–Mayer: improved models for short-range repulsion in ab initio force fields. J. Chem. Theory Comput..

[20] Smith JS (2019). Approaching coupled cluster accuracy with a general-purpose neural network potential through transfer learning. Nat. Commun..

[21] von Lilienfeld OA, Müller K-R, Tkatchenko A (2020). Exploring chemical compound space with quantum-based machine learning. Nat. Rev. Chem..

[22] Stone, A. *The Theory of Intermolecular Forces*. (OUP Oxford, 2013).

[23] Ceriotti M (2016). Nuclear quantum effects in water and aqueous systems: experiment, theory, and current challenges. Chem. Rev..

[24] Horta BAC (2016). A GROMOS-compatible force field for small organic molecules in the condensed phase: the 2016H66 parameter set. J. Chem. Theory Comput..

[25] Bash PA, Singh UC, Langridge R, Kollman PA (1987). Free energy calculations by computer simulation. Science.

[26] Levitt M (1976). A simplified representation of protein conformations for rapid simulation of protein folding. J. Mol. Biol..

[27] Allen, M. P. & Tildesley, D. J. *Computer Simulation of Liquids* (Oxford University Press, 2017).

[28] Ewald PPDie (1921). Berechnung optischer und elektrostatischer Gitterpotentiale. Ann. Phys..

[29] Darden T, York D, Pedersen L (1993). Particle mesh Ewald: an N⋅log(N) method for Ewald sums in large systems. J. Chem. Phys..

[30] Martyna GJ, Klein ML, Tuckerman M (1992). Nosé–Hoover chains: the canonical ensemble via continuous dynamics. J. Chem. Phys..

[31] Martyna GJ, Tuckerman ME, Tobias DJ, Klein ML (1996). Explicit reversible integrators for extended systems dynamics. Mol. Phys..

[32] Tuckerman ME, Berne BJ, Martyna GJ, Klein ML (1993). Efficient molecular dynamics and hybrid Monte Carlo algorithms for path integrals. J. Chem. Phys..

[33] Feynman, R. P., Hibbs, A. R. & Styer, D. F. *Quantum Mechanics and Path Integrals* (Courier Corporation, 2010).

[34] Martyna GJ, Hughes A, Tuckerman ME (1999). Molecular dynamics algorithms for path integrals at constant pressure. J. Chem. Phys..

[35] Balog E, Hughes AL, Martyna GJ (2000). Constant pressure path integral molecular dynamics studies of quantum effects in the liquid state properties of n-alkanes. J. Chem. Phys..

[36] Grimme S (2014). A general quantum mechanically derived force field (QMDFF) for molecules and condensed phase simulations. J. Chem. Theory Comput..

[37] Mobley DL, Bayly CI, Cooper MD, Shirts MR, Dill KA (2009). Small molecule hydration free energies in explicit solvent: an extensive test of fixed-charge atomistic simulations. J. Chem. Theory Comput..

[38] Weinreich J, Browning NJ, von Lilienfeld OA (2021). Machine learning of free energies in chemical compound space using ensemble representations: reaching experimental uncertainty for solvation. J. Chem. Phys..

[39] Ehlert S, Stahn M, Spicher S, Grimme S (2021). Robust and efficient implicit solvation model for fast semiempirical methods. J. Chem. Theory Comput..

