# Supplementary material for: Accurate determination of solvation free energies of neutral organic compounds from first principles
Source: Nat Commun. 2022 Jan 20;13:414. doi: 10.1038/s41467-022-28041-0 (PMC8776904; doi:10.1038/s41467-022-28041-0)
Supplement: Supplementary file 1 — Supplementary Information [file 41467_2022_28041_MOESM1_ESM.pdf]

Supplementary Information

Accurate determination of solvation free energies of neutral organic compounds from first principles

L. Pereyaslavets et al.

1 Supplementary methods

Quantum Mechanical details

The total dimer energies are calculated with silver standard [1] i.e. MP2/CBS, calculated with Helgaker cubic extrapolation [2] from aug-cc-pVTZ->aug-cc-pVQZ + CCSD(T)/aug-cc-pVDZ - MP2/aug-cc-pVDZ. To improve transferability and ease our optimization we use DFT-SAPT[3–5] decomposition with δHF correction [4] at aug-cc-pVTZ level with PBE0 functional asymptotically corrected[4]. We employ four parts of DFT-SAPT decomposition which have corresponding manifestation in our FF : ES (electrostatic E1pol), EX (exchange-repulsion E1exch), IND (induction, E2ind + E2ind-exch + δHF), DS (dispersion, E2disp+E2disp-exch + Esilver-standard - DFT-SAPTtotal_energy). The dispersion term accumulates all disagreements between total energy described by the "silver standard" and DFT-SAPT+δHF energies.

Force Field description

A significant effort has been undertaken in the past few decades to develop QM parametrized force fields with applications targeted towards bio and material science [6–11].
Our model - the ARROW Force Field or ARROW FF - has the following features: The two-body non-bonded interactions are composed of electrostatic, exchange-repulsion and dispersion terms. The electrostatic and exchange-repulsion terms are multipolar, and their radial dependence is a Slater-like [12,13] exponential so that they're better able to describe charge penetration and delocalization. The dispersion term is conventionally represented by a spherical, 2 term (C6 and C8), Tang-Toennies-damped interaction [14]. Many-body effects are modeled by anisotropic atomic polarizable dipoles interacting with the electrostatic term and with each other and iterated to self-consistent field (SCF) convergence on every non-bonded step. There are no explicit three-body terms [e.g., Axilrod–Teller [15]], and we do not include charge flux. The intermolecular parameters of ARROW FF are determined by agreements with QM values of dimer and multimer energies, electrostatic potentials, and multipole moments of monomers. To aid transferability, we also attempt to match the individual components to their corresponding QM counterparts, in addition to reproducing the total energy. The bonded interactions are fitted to monomer QM energies computed at the df-MP2/aug-cc-pVTZ level of theory, as is standard practice in the field.

Force field functional form of ARROW FF

Arrow FF follows the functional form published in [13].

A) ARROW Charge Density function

In the ARROW FF, the charge density of the electron cloud of atom '*a*' located at position R_a is written as:

$$\rho_a(\mathbf{r}) = \hat{D}_a \frac{q_a}{8\pi w_A^3} \exp\left(-\left|\mathbf{r} - \mathbf{R}_a\right| / w_A\right), \tag{A1}$$

where q_a is the cloud charge and w_A is the ARROW atom-type parameter that characterizes the cloud size. We use the capital letters '*A*', '*B*', ... to denote the ARROW atom- types respectively for atoms 'a', 'b', ...; similar notations are used for bond, bend and torsion atoms types (e.g. '*AB*' denotes the ARROW bond type for the pair of bonded atoms 'a' and 'b').

The cloud charge is written in the form:

$$q_a = -Z_A + \sum_{b \in \{a\}} q_{BA} \tag{A2}$$

where Z_A is the charge of the "nucleus" for the atom type '*A*', {a} denotes the set of atoms chemically bonded to atom '*a*' and q_{BA} is the ARROW bond charge transfer parameters (thus the equality $q_{AB} = -q_{BA}$ providing for charge conservation).

Differential operator $\hat{D}_a$

$$\hat{D}_a \equiv 1 + \mathbf{t}_a \frac{\partial}{\partial \mathbf{R}_a} + \frac{1}{2}\left(\frac{\partial}{\partial \mathbf{R}_a}\right)^T \boldsymbol{\omega}_a \frac{\partial}{\partial \mathbf{R}_a} \tag{A3}$$

where dimensionless vector t_a and 3×3 dimensionless tensor ω_a introduce *p and d* type density anisotropy, respectively. They are related to the local atomic dipole $\mathbf{P}_a$ and atomic quadrupole $\mathbf{Q}_a$ as:

$$\begin{aligned} \mathbf{P}_a &= q_a \mathbf{t}_a, \\ \mathbf{Q}_a &= q_a \boldsymbol{\omega}_a \end{aligned} \tag{A4}$$

Vector $\mathbf{t}_a$ is a sum of permanent and induced vectors:

$$\mathbf{t}_a = \mathbf{t}_a^{per} + \mathbf{t}_a^{ind} \tag{A5}$$

The induced part is written as

$$\mathbf{t}_a^{ind} = t_A^{\max} \boldsymbol{\tau}_a \tag{A6}$$

where t^{max}_A is the ARROW atom-type parameter and $\boldsymbol{\tau}_a$ is the dimensionless vector representing the ARROW dynamic variable, its length being confined within interval (0, 1) by the restraint

potential U_{IN}. The induced variable is iterated to convergence (1E-6 kcal/mol/A) of the electrostatic and exchange forces in an SCF (self-consistent-field) manner as is common in polarizable Force Fields. In this work we do not employ Extended Largrangian techniques but iterate the dipoles explicitly to convergence.

The permanent part in Eq. (A5) is written as:

$$\mathbf{t}_a^{per} = \sum_{b \in \{a\}} t_{AB} \mathbf{n}_{ab} \tag{A7}$$

where $\mathbf{n}_{ab}$ is the unit vector, $\mathbf{n}_{ab} = R_{ab} /| R_{ab} |$ and R_{ab} is the vector directed from atom ‘a’ to neighboring atom ‘b’ and t_{AB} is the ARROW bond-type parameter associated with permanent 'chemical' polarization or “charge shift” along the bond 'ab ' (in general $\mathbf{t}_{AB} \neq \mathbf{t}_{BA}$).

As for the local quadrupole tensor, its components are calculated as:

$$(\omega_a)_{\alpha\beta} = 2\sum_{b\in\{a\}} Q_{AB}\left((n_{ab})_\alpha (n_{ab})_\beta - \frac{\delta_{\alpha\beta}}{3}\right) \tag{A8}$$

where α,β = x, y, z and Q_{AB} are the ARROW bond-type parameters.

The atom “nucleus” (proportional to Dirac’s delta-function) can be formally considered as the limiting case of Eq. (A1) replacing q_a by Z_A , dropping operator $\hat{D}_a$ and formally approaching zero by the width parameter.

B) Non-Valence Interactions

Electrostatic potential

Based on representation of Equation (A1) the potential φ_{ab} of electrostatic interaction of two unit charge distributions ‘*a*’ and ‘*b*’ separated by a distance $R_{ab} = |R_b – R_a|$ can be represented in the form:

$$\varphi_{ab}(\mathbf{R}_{ab}, \mathbf{t}_a, \mathbf{t}_b) = \hat{D}_a \hat{D}_b \varphi_{ab}^{(0)}(R_{ab}) \tag{B1}$$

$$\varphi_{ab}^{(0)}(r) = \frac{1}{r}\left[1 - f(w_A, w_B)e^{-r/w_A} - f(w_B, w_A)e^{-r/w_B}\right]$$

$$f(u,v) \equiv \frac{u^4(3v^2 - u^2)}{(v^2 - u^2)^3} + \frac{u^3}{2(v^2 - u^2)^2} r. \qquad \text{(B2)}$$

Expanding equation (B1) one gets:

$$\varphi_{ab}(\mathbf{R}_{ab}, \mathbf{t}_a, \mathbf{t}_b) = \sum_{k=0}^{4} F_{ab}^{(k)}(\mathbf{R}_{ab}, \mathbf{t}_a, \mathbf{t}_b)\varphi_{ab}^{(k)}(R_{ab}) \qquad \text{(B3)}$$

where *F's* are the algebraic factors defined as:

$$\begin{aligned}
F_{ab}^{(0)} &= 1,\\
F_{ab}^{(1)} &= (\mathbf{t}_b\mathbf{R}_{ab}) - (\mathbf{t}_a\mathbf{R}_{ab}) - (\mathbf{t}_a\mathbf{t}_b),\\
F_{ab}^{(2)} &= -(\mathbf{t}_a\mathbf{R})(\mathbf{t}_b\mathbf{R}) + \frac{1}{2}\mathrm{Sp}\,\boldsymbol{\omega}_a\boldsymbol{\omega}_b\\
&\quad + \frac{1}{2}(\mathbf{R}^+\boldsymbol{\omega}_a\mathbf{R}) + \frac{1}{2}(\mathbf{R}^+\boldsymbol{\omega}_b\mathbf{R}) + (\mathbf{R}^+\boldsymbol{\omega}_a\mathbf{t}_b) - (\mathbf{R}^+\boldsymbol{\omega}_b\mathbf{t}_a),\\
F_{ab}^{(3)} &= \frac{1}{2}(\mathbf{t}_b\mathbf{R})(\mathbf{R}^+\boldsymbol{\omega}_a\mathbf{R}) - \frac{1}{2}(\mathbf{t}_a\mathbf{R})(\mathbf{R}^+\boldsymbol{\omega}_b\mathbf{R}) + (\mathbf{R}^+\boldsymbol{\omega}_a\boldsymbol{\omega}_b\mathbf{R}),\\
F_{ab}^{(4)} &= \frac{1}{4}(\mathbf{R}^+\boldsymbol{\omega}_a\mathbf{R})(\mathbf{R}^+\boldsymbol{\omega}_b\mathbf{R}).
\end{aligned} \qquad \text{(B4)}$$

and potentials $\varphi^{\{k\}}_{ab}$ satisfy the recurrence relations:

$$\varphi_{ab}^{(k+1)}(R) = \frac{1}{R}\frac{d}{dR}\varphi_{ab}^{(k)}(R), \qquad k = 0,1,... \qquad \text{(B5)}$$

The ES potential U^{ES} between the two clouds is written as:

$$U_{ab}^{ES}(\mathbf{R}_{ab}) = 332.064 \cdot q_a q_b \varphi_{ab}(\mathbf{R}_{ab})$$

The potentials are in kcal/mol, the components of all vectors R_{ab} and t are in Å, charges are in a.u. This formula is also valid for "nucleus"-cloud and "nucleus"-"nucleus" interactions by replacing the cloud charges with the nucleus charges, nullifying vectors **t** and tensor **ω** for the core, with the corresponding width parameter(s) formally approaching zero.

Exchange potential

The multipolar EX potential U^{EX} is written similarly to that of ES:

$$U_{ab}^{EX} = 332.064 \cdot C_A^{EX} C_B^{EX} \chi_{ab}(\mathbf{R}_{ab}) \tag{B6}$$

where $C^{EX}{}_A$, $C^{EX}{}_B$ are the force parameters for atom types '*A*' and '*B*', and

$$\chi_{ab}(\mathbf{R}_{ab}, \mathbf{t}_a, \mathbf{t}_b) = \sum_{k=0}^{4} F_{ab}^{(k)}(\mathbf{R}_{ab}, \mathbf{t}_a, \mathbf{t}_b) \chi_{ab}^{(k)}(R_{ab}) \tag{B7}$$

The algebraic factors *F* were defined in Eq. (B4), potentials $\chi^{\{k\}}{}_{ab}(R_{ab})$ satisfy the recurrence relations in Eq. (B5)

$$\chi_{ab}^{(0)}(r) \equiv u_A u_B \frac{e^{-r/u_B} - e^{-r/u_A}}{(u_B - u_A) r} \tag{B8}$$

where u_A and u_B are the EX width parameters that generally differ from ES parameters w_A and w_B. Again, $\chi_{ab}{}^{(0)}$ represents a functional form of the exchange potential.

Dispersion potential

$$U_{ab}^{DS} = -332.064 \cdot \left(C_A^{DS6} C_B^{DS6} \psi_6(R_{ab}) + C_A^{DS8} C_B^{DS8} \psi_8(R_{ab}) \right) \tag{B9}$$

where C^{DS6} , C^{DS8} are the force constants and ψ's are the Tang-Toennies's functions [14]:

$$\psi_n(r) = r^{-n} \left[1 - e^{-2r/(v_A+v_B)} \sum_{k=1}^{n} \frac{1}{k!} \left(\frac{2r}{v_A + v_B} \right)^k \right] \tag{B10}$$

v_A and v_B being the dispersion width parameters that generally differ from those for electrostatics and exchange.

Induction potential

The corresponding anharmonic restraint potential for the atom 'a' being represented as:

$$U_a^{IN}(\boldsymbol{\tau}_a) = 2240.88 \cdot \frac{(q_a t_A^{\max})^2}{\alpha_A M_a} \frac{\tau_a^2 + \sum_{b \in \{a\}} s_{AB} (\boldsymbol{\tau}_a \mathbf{n}_{ab})^2}{1 + \sqrt{1 - (\boldsymbol{\tau}_a)^2}}, \qquad M_a = 1 + \tfrac{1}{3} \sum_{b \in \{a\}} s_{AB} \tag{B11}$$

where α_A is the ARROW parameter characterizing polarizability prescribed to atom-type *A* while bond-type parameters s_{AB} describe the anisotropy of the restraint potential (note that generally

$s_{AB} \neq s_{BA}$), the vector τ_a was introduced in Eq. (A6). Note that the force due to potential in Eq. (B11) approaches infinity as τ_a approaches 1. This peculiarity of the ARROW restraint potential avoids the polarization catastrophe and provides the existence of a solution of the problem of optimization of electron density in any physically reasonable external field.

C) Induced dipoles implementation

It is clearly seen from equations (B1-B11) that the system depends on polarizable part of clouds $\mathbf{t}_a^{ind} = t_A^{\max} \boldsymbol{\tau}_a$ that responds to electrostatic fields of other cloud charges, “nuclei”, cloud dipoles, cloud quadrupoles as well as other polarizable clouds in the system. All these equations of self-consistent polarizable clouds can be represented as typical quadratic form of induced polarizable shifts or dipoles $E = E_{\tau-independent} + \sum \tau_a \tau_b + \sum C_a \tau_a + \sum C_b \tau_b$. Such induced dipole approach to polarizable force fields is commonly known how to be solved [16]. This quadratic form is optimized with steepest descent with respect to polarizable clouds vector τ_a at every step of molecular dynamics until it reaches the required small force threshold which is typically about 1E-6 kcal/mol/A.

D) Valence Interactions

Similar to the MMFF-94 force field, the Hamiltonian of valence interaction is given in the form:

$$H = H^{BS} + H^{AB} + H^{StBn} + H^{OOP} + H^{TORS} \tag{C1}$$

where the terms correspond respectively to bond stretching (*BS*), angle bending (*AB*), stretch-bend (*St-Bn*), out-of-plane bending (*OOP*), and torsion interactions (*TORS*).

The bond stretching component is of the form:

$$H^{BS} = \frac{1}{2} \sum k_{IJ}^{(b)} \Delta r_{ij}^{\ 2} \left(1 + c_b \Delta r_{ij} + \tfrac{7}{12} c_b^{\ 2} \Delta r_{ij}^{\ 2}\right) \tag{C2}$$

where

$$\Delta r_{ij} = \left|\mathbf{r}_i - \mathbf{r}_j\right| - r_{IJ}^{(0)} \tag{C3}$$

In Eq. (C2), the sum is over all the bond pairs, I and J are the indices of the atomic types ascribed respectively to atoms *i* and *j*, $r_{IJ}^{(0)}$ and $k_{IJ}^{(b)}$ are respectively the equilibrium (or reference) bond length and the bond force constant determined by the atomic type pair and the specific bond type index, and c_b is a model parameter.

The angle bending term is given by:

$$H^{AB} = \frac{1}{2}\sum k_{IJK}^{(a)} \Delta\varphi_{ijk}{}^{2} \left(1 + c_a \Delta\varphi_{ijk}\right) \tag{C4}$$

where the summation is performed over all bend triplets i-j, j-k, the indices I, J and K corresponding to the atomic types ascribed respectively to atoms *i*, *j* and *k*. Other notations are:

$$\Delta\varphi_{ijk} = \varphi_{ijk} - \varphi_{IJK}^{(0)} \tag{C5}$$

where φ_{ijk} is the angle between i-j and k-j bonds, $\varphi_{IJK}{}^{\{0\}}$ and $k_{IJK}{}^{\{a\}}$ are the equilibrium (or reference) angle and the force constant determined by the atomic type triplet and the specific angle type index, and c_a is a model parameter.

Stretch-bend interaction is given by:

$$H^{St-Bn} = \sum \Delta\varphi_{ijk} \left(k_{IJK}^{(ba)} \Delta r_{ij} + k_{KJI}^{(ba)} \Delta r_{kj}\right) \tag{C6}$$

where the summation is similar to Eq. (C4). The bond length and angle deviations, Δr_{ij}, Δr_{kj} and $\Delta\varphi_{ijk}$ are defined by Eqs. (C3) and (C5), $k^{(ba)}{}_{IJK}$ and $k^{(ba)}{}_{KJI}$ are the force constants for the interaction of ij and kj bond-angle, respectively. These constants are determined by the atomic type triplet and the specific stretch-bend type index.

Out-of-plane interaction is given by:

$$H^{OOP} = \frac{1}{2}\sum k_{IJKL}^{(oop)} \left(\Delta\chi_{ikl}{}^{2} + \Delta\chi_{kli}{}^{2} + \Delta\chi_{lik}{}^{2}\right) \tag{C7}$$

where the sum is over all bond tetrahedrals i-j-k-l with the 3-bond central (or vertex) atom *j* and the bonds i-j, k-j, l-j, the indices I, J, K and L correspond to the atomic types ascribed respectively to atoms *i*, *j*, *k*, and *l*. The angle deviation $\Delta\chi_{ikl}$ is defined as the angle between the vector ($\boldsymbol{r}_i - \boldsymbol{r}_j$) and the plane defined by vectors ($\boldsymbol{r}_k - \boldsymbol{r}_l$) and ($\boldsymbol{r}_l - \boldsymbol{r}_j$); that is

$$\sin\left(\Delta\chi_{ikl}\right) = \frac{\mathbf{n}_{ij} \cdot \left(\mathbf{n}_{kj} \times \mathbf{n}_{lj}\right)}{\left|\mathbf{n}_{kj} \times \mathbf{n}_{lj}\right|} \tag{C8}$$

where

$$\mathbf{n}_{aj} = \left(\mathbf{r}_a - \mathbf{r}_j\right) / \left|\mathbf{r}_a - \mathbf{r}_j\right|, \quad a = i, k, l \tag{C9}$$

The force constant k_{IJKL} is determined by the atomic type quartet IJKL.

The torsion component is of the form

$$H^{TORS} = \frac{1}{2}\sum\left[V_{IJKL}^{(1)}\left(1+\cos\phi\right)+V_{IJKL}^{(2)}\left(1-\cos 2\phi\right)+V_{IJKL}^{(3)}\left(1+\cos 3\phi\right)\right] \tag{C10}$$

where the sum is over all bond quartets i-j , j-k , k-l , the indices I, J, K, and L correspond to the atomic types ascribed respectively to atoms *i*, *j*, *k*, and *l*. *ø* is a standard torsion angle defined as the angle between the planes i-j-k and j-k-l, given by:

$$\cos\phi = \mathbf{m}_{ij} \cdot \mathbf{m}_{lk} \tag{C11}$$

where

$$\mathbf{m}_{ij} = \frac{\mathbf{n}_{ij} - \mathbf{n}_{jk}\left(\mathbf{n}_{jk} \cdot \mathbf{n}_{ij}\right)}{\sqrt{1-\left(\mathbf{n}_{ij} \cdot \mathbf{n}_{jk}\right)^2}}, \qquad \mathbf{m}_{kl} = \frac{\mathbf{n}_{kl} - \mathbf{n}_{jk}\left(\mathbf{n}_{jk} \cdot \mathbf{n}_{kl}\right)}{\sqrt{1-\left(\mathbf{n}_{kl} \cdot \mathbf{n}_{jk}\right)^2}} \tag{C12}$$

The force constants V_{IJKL} are determined by the atomic type quartet and two specific indices, the first relating to the type of the central bond j-k , while the second one relates to the hybridization state of atoms *j* and *k*.

Parameter fitting

Monomer, dimer, and multimer QM data of high quality is used for fitting the model. From the monomer calculations we use the ES potential map as well as polarizability and its related properties. Some initial guesses for the ES serve as hyperbolic regularizers as in the original RESP approach. The final fit of all parameters together can be done is a well-established art. We currently prefer the stochastic Covariance matrix adaptation evolution strategy (CMA-ES). To aid in clarity we include a flowchart of the parametrization process, see Supplementary Figure 1.

Pseudo code for parameterization and simulation used in determination of solvation free energies

```
1. Assign_atom_types (molecule) {
        for (atoms in molecule):
                obtain_molecular_neighborhood(atom)
                assign_chemical_atom_type(atom)
        }

2.  Obtain_QM_data(monomers, dimers, multimers) {  #we use Molpro
        geometric_optimization(molecule)
        compute_electrostatics(molecule)
        compute_polarization(molecule)
        decompose_onto_atom_centers() # es potential, moments and polarizability
```

```
        dimer_qm_calculations(molecule1, molecule2) {
                molecule1_molecule1_calcs  (molecule1, molecule1)
                molecule1_H2O_calcs (molecule1, H2O)
                molecule1_Me_calcs (molecule1, CH4) # optional
        }

3. Obtain_FF_params(train_set, QM_data) {
        # QM data = [dispersion, electrostatics, exchange, induction,
                multimer energies, multipole moments]
        input_data(train_set_coords, QM_data)
        set_initial_param_guess()
        for (iteration=1; convergence=sufficient; i++):
                #CMA-ES or stochastic gradient descent
                minimize_qm_ff_deviation (new_param_guess, train_set, FF_result)
        }

# not in loop because rarely required
4. if (convergence > desired_convergence) {
        change_typification(molecule) #e.g. C3H8 may acquire 2 C types
        goto (3.) # Obtain_FF_params again
}



5. Run_MD(coords, FF_params, num_PIMD_beads) {
        Setup(solute, solvent, box_params, barostat, thermostat, temp, press) {
                place_molecules()
                thermalize_system()
                run_alchemical_simulation(time) # 1ns in this case
                # in the distribution CPU runs ./run_cpu.sh and GPU runs ./run_gpu.sh
}

6. MD_analysis(md_output) {
# scripts (bar.sh, fermiBAR.m, out2mat.m, runBAR_noneven.m)
        compute_BAR_solvation(md_output)
        compute_TI_solvation(md_output)
        if (bar_result != ti_result):  #does not happen in solvation
                increase_num_ti_points()
                goto 5.
}
```

Ethanol-water dimers as a function of closest distance

The FF:QM errors for ethanol-water dimers as a function of closest distance are shown in Supplementary Figure 2. The FF:QM faithfulness becomes worse in the region of close approach and high electron overlap where the electron densities and corresponding integrals have large values and derivatives. The worst low-energy dimer (E - -4.5 kcal/mol, ERR = 0.9 kcal/mol) is shown in the figure. This configuration can be better described by going to a higher

(L=3,4) level of the atomic multipole expansion and/or by including multiple Slater exponents, but we chose our truncations of L=2 and one exponent for reasons of computational speed and parameter complexity. The current work shows this is a sufficient cutoff for having a good QM:FF agreement for the overall ensemble of possible configurations.

Many-body interactions: specific case ethanol-water multimer

Multimers of ethanol with 2,3,and 4 water molecules were extracted from molecular dynamics simulations with the ARROW force field. Geometries of those multimers were optimized with the mp2/cc-pVTZ method. Nonadditive energies are full nonbonded energy of MP2/CBS (aug-cc-pVTZ->aug-cc-pVQZ extrapolated) minus energies of dimers nonbonded energy. The non-additive many-body error for multimers of ethanol with 2,3,4 water molecules correspondingly plotted versus their total QM intermolecular energy are shown in Supplementary Figure 3. All the many-body errors are below 1 kcal/mol, or below ~2% of total energy.

Double logarithmic Force field training-learning curves: n-methyl-acetamide, acetamide and water.

As expected for 'formula'- based functional forms, the predictability of the model saturates at its maximum achievable accuracy. In contrast, ML models can theoretically achieve perfect accuracy, but the training data requirements are linear in log-log (accuracy vs training size) space. Such a double logarithm learning plot for amides and water is shown in Supplementary Figure 4.

Comparison with GFN-FF on Benchmarks S22 and S66.

We have validated our FF on commonly known S22[17] and S66[18] benchmarks and compared it with reported values for GFN-FF. For proper comparison we have recalculated the QM energies for known geometries at our silver-like standard (see quantum mechanical). The geometries were taken from http://www.begdb.org website. The resulting MAEs for the dimer datasets as predicted by ARROW FF and GFN-FF are presented in Supplementary Table 1.The dimer energetics as predicted by the ARROW FF for the S22 and S66 dataset are tabulated in Supplementary Data 1f. As GFN-FF currently enables solvation calculations via a tuned implicit model only, we can only attempt to estimate the resulting error of solvation for an explicit water calculation. Propagating the MAE forward to the final solvation energetics: a molecule surrounded by 4-6 molecules would be off by 2-3*0.8 kcal/mol = 1.6-2.4 kcal/mol error of interaction of that molecule with its neighbourhood. This is the best case scenario and assumes a zero mean error (recall that in DFT itself the mean error is definitely not zero and varies for different types of molecules). Our major emphasis and achievement is accuracy + general coverage suitable for biomolecular simulations (e.g. for water and solutes is about 0.2 kcal/mol MAE which will roughly result in 2-3*0.2 = 0.4-0.6 kcal/mol error of interaction of that molecule with neighbourhood. Such an accuracy has only been achieved previously by groups describing

one (H2O mostly, e.g. Medders et al. 2014) or a couple of chemical species (Veit et al. 2019, Deringer et al. 2020) while we have achieved it on all major neutral chemical groups and solvents.

Simulation details and protocols

The molecules were placed in a water or cyclohexane box of size 32 X 32 X 32 Å^3. Particle-Mesh Ewald (PME) algorithm [19] was used to compute the electrostatic interactions. A 32 X 32 X 32 grid mesh and 5th power spline interpolation order were used to compute the inverse PME sum. 9 Å cutoffs were used to compute the direct PME sum as well as the exchange and dispersion interactions. Bulk corrections were applied to account for distance cut-off of dispersion interactions. Solvation free energies were computed by decoupling the interactions between the solute and the solvent molecules. Electrostatic, exchange-repulsion and dispersion interactions were switched off simultaneously using a lambda-dependent scaling (a scaling factor power = 2 was used) and a soft-coring algorithm (maximal soft-coring radius = 1.5 Å, soft-coring radius scaling factor power = 1). 15 lambda points unequally spaced (λ = 0, 0.05, 0.1, 0.2, 0.3, 0.4, 0.5, 0.6, 0.7, 0.75, 0.8, 0.85, 0.9, 0.95, 1.0) were used to decouple the solute-solvent intermolecular interactions.

Energy minimization (10,000 steps) using the steepest descent algorithm was initially performed on all the simulation systems. This was followed by a 50 ps equilibration and 1 ns production runs in the *isothermal-isobaric* ensemble (*NPT*). The temperature was maintained at 298 K using a Nose-Hoover thermostat [20] (chain length = 6, relaxation time = 1 ps). Pressure was maintained at 1 atm using a MTTK barostat [21] (relaxation time = 5 ps). A Multiple Time Step (MTS) algorithm [22] was used to integrate the equations of motion both for the classical and path-integral runs. Bonded and PIMD [23–25] beads interactions were integrated with the time step of 0.125 fs, while intermolecular interactions were integrated with a time step of 2 fs. Our PIMD implementation follows classical work of Tuckerman and Martyna [23–25] which is applied to all atoms in the system. All the calculations were performed with our in-house ARBALEST program [26,27]. The intra- and intermolecular energies and forces are computed using NVIDIA GPUs, and integration of the extended equations of motion is done on the CPU.

We compute the solvation free energies using both the Bennett acceptance ratio (BAR) [28] and thermodynamic integration (TI)[29] method with <dH/dλ> values interpolated by cubic splines. A typical **<**dH/dλ> dependence for MD and PIMD (8 replicas) on desolvation coordinate λ (anthracene in water) is presented in Supplementary Figure 5a. For a 1 ns trajectory and for different λ states the estimated statistical error of computed free energy of solvation is about 0.2 kcal/mol, see Supplementary Figure 5b. This value is close to the observed RMS errors for computed solvation △G vs experiment and to the estimated error of experimental solvation △G values. Based on the described protocol we determined the free energies of solvation for various functional groups using classical and path-integral methods. The data for free energies of hydration, solvation and partition coefficients for the various functional groups, and amino acid analogues can be found in Supplementary Data 1a, 1b, respectively. As stated in the main text, we also make comparisons of the hydration free energies as predicted by ARROW, GAFF, and AMOEBA force fields, see Supplementary Data 1c. Additionally we also report the

computations of free energies of hydration performed independently by our academic collaborations, see Supplementary Data 1d. Time required for computation of 1 ns molecular dynamics solvation simulation for each window using ARROW FF based on classical and path-integral methods are tabulated in Supplementary Table 3.

Comparison to Implicit solvent models and Machine learning models

In Supplementary Data 1e, we tabulate and compare the free energy of hydration predictions of various functional group molecules based on ARROW FF PIMD to that of the implicit solvent models namely Solvation Model Dielectric (SMD), Polarizable Continuum Model (PCM), and COSMO/COSMO-RS. Due to unavailability of free energy data for the original COSMO model, we computed the free energies of solvation for our molecule dataset using MolPro *ver.* 2012 (gcosmo, epsilon=80.0,df-ks, *B3LYP*). The MAE for COSMO (computed using MolPro *ver.* 2012) is 2.5 kcal/mol which is in reasonable agreement with the MAE of ~2 kcal/mol for COSMO as reported by Weinreich et al. [30]. The comparisons to other implicit solvent models such as SMD, PCM and COSMO-RS are limited to the overlap of the molecules computed using ARROW. The free energies of hydration as predicted by these implicit solvent models (SMD, PCM) were obtained completely from published literature [31]. For these subset of compounds, as one can see in Supplementary Data 1e, the MAE's for the SMD, PCM are 0.61 kcal/mol and 1.49 kcal/mol, respectively. We also collected the free energies of hydration as determined by COSMO-RS [32] and for our dataset we found the MAE to be 0.42 kcal/mol. We also make comparisons to the free energies of hydration for the various compounds as predicted by the Geometry, Frequency, Non-covalent Force field (GFN-FF) of Grimme et al. [33]. The MAE for the GFN-FF predictions of the current dataset is 1.72 kcal/mol which is a reasonable and expected number for models treating water as an implicit solvent. Supplementary Table 3 summarizes the MAE's of free energies of hydration predicted by the various atomistic and implicit solvent models. To put all numbers into perspective, we have added a comparison of the MAE's to an optimized zero-model with only one number for all species in our dataset $\delta G_{predicted}$ = -3.5 kcal/mol. It results in a MAE=2.467 kcal/mol which is useful when comparing the different models, e.g. for COSMO, improvement is 0.986 times and for ARROW FF improvement it is 11.2 times with respect to the zero-model ($\delta G_{predicted}$ =-3.5 kcal/mol).

Machine learning models targeted towards free energy determination have become a popular avenue in the past few years [30,34]. Based on an overlapping dataset of free energy computations of neutral organic molecules, we found that the MAE for Free Energy Machine Learning Model (FML) was 0.50 kcal/mol. Our computed MAE's for the free energies of hydration on our subset of neutral organic molecules dataset based on these implicit solvent models and the machine learning model-FML are consistent with the MAE's reported by Weinreich et al. [30]. The MAE's for these various implicit solvent models and FML are tabulated in Supplementary Table 3. A comparative plot for such models and ARROW-FF is shown in Supplementary Figure 6.

PIMD bead convergence on free energy of solvation results and radial distribution functions for water

To check the bead convergence on the free energies of solvation, we computed the free energies of hydration and solvation for water, ethanol, and cyclohexane as a function of PIMD bead sizes (1,2,4,8,16,and 32), see Supplementary Figure 7. To check the convergence of bead size on the structural properties, we compute the hydrogen-hydrogen (H-H), hydrogen-oxygen (H-O) and oxygen-oxygen (O-O) pair-wise RDF of water as a function of bead size and such results can be seen in Supplementary Figure 8.

2 Supplementary Tables

Supplementary Table 1: Comparison of MAE's for s22 and s66 dataset. MAE of the noncovalent interaction energies computed with ARROW and GFN-FF taken from the paper [11] for known benchmark sets. For ARROW FF, the QM reference numbers were calculated using the silver-standard. The values are given in kcal mol−1 .

Benchmark set	ARROW FF	GFN-FF
s22	0.207	0.89
s66	0.194	0.76

Supplementary Table 2: Computation times for solvation on CPU and GPU. Time required for computation of 1 ns molecular dynamics solvation simulation for each window using ARROW FF based on classical and path-integral methods. As the emphasis in this work is on accuracy, these timings do not employ the various speed-up techniques that may result in energy errors >0.1 kcal/mol. e.g. centroid RPMID was not used in these runs, the time-steps were highly conservatively picked, etc.

GPU/CPU type	MD	PIMD 8
GeForce RTX 2080 TI + 2 CPU cores	~2 hours	~16-18 hours
CPU 8 cores	~10 hours	~78 hours

Supplementary Table 3: Comparison of MAE's for various implicit solvent models, FML, and ARROW FF. Mean absolute error (MAE) for the free energies of hydration of various organic molecules as predicted by the Force Fields and implicit solvent models.

Force Field/Model	Free energy of hydration MAE (kcal/mol)
ARROW-FF	0.22
GAFF	0.88
AMOEBA	0.76
COSMO-MolPro	2.50
SMD	0.61
PCM	1.49
FML	0.50
COSMO-RS	0.42
GFN-FF δE(ALPB)	1.72
GFN-FF δG(ALPB)	1.72
Zero-model (δG=-3.5 kcal/mol)	2.47

Supplementary Table 4: **Bead convergence on free energies of solvation**. Free energies of hydration and solvation (kcal/mol) as a function of path-integral beads.

hydration	Expt	PIMD1	PIMD2	PIMD4	PIMD8	PIMD16	PIMD32
water	-6.30	-6.82	-6.34	-6.20	-6.14	-6.08	-6.04
ethanol	-5.01	-5.75	-5.37	-5.18	-4.94	-4.87	-4.84

solvation CHEX	Expt	PIMD1	PIMD2	PIMD4	PIMD8	PIMD16	PIMD32
cyclohexane	-4.42	-4.23	-4.10	-4.02	-3.94	-3.89	-3.86
ethanol	-2.59	-2.65	-2.55	-2.48	-2.43	-2.40	-2.35

3 Supplementary Figures

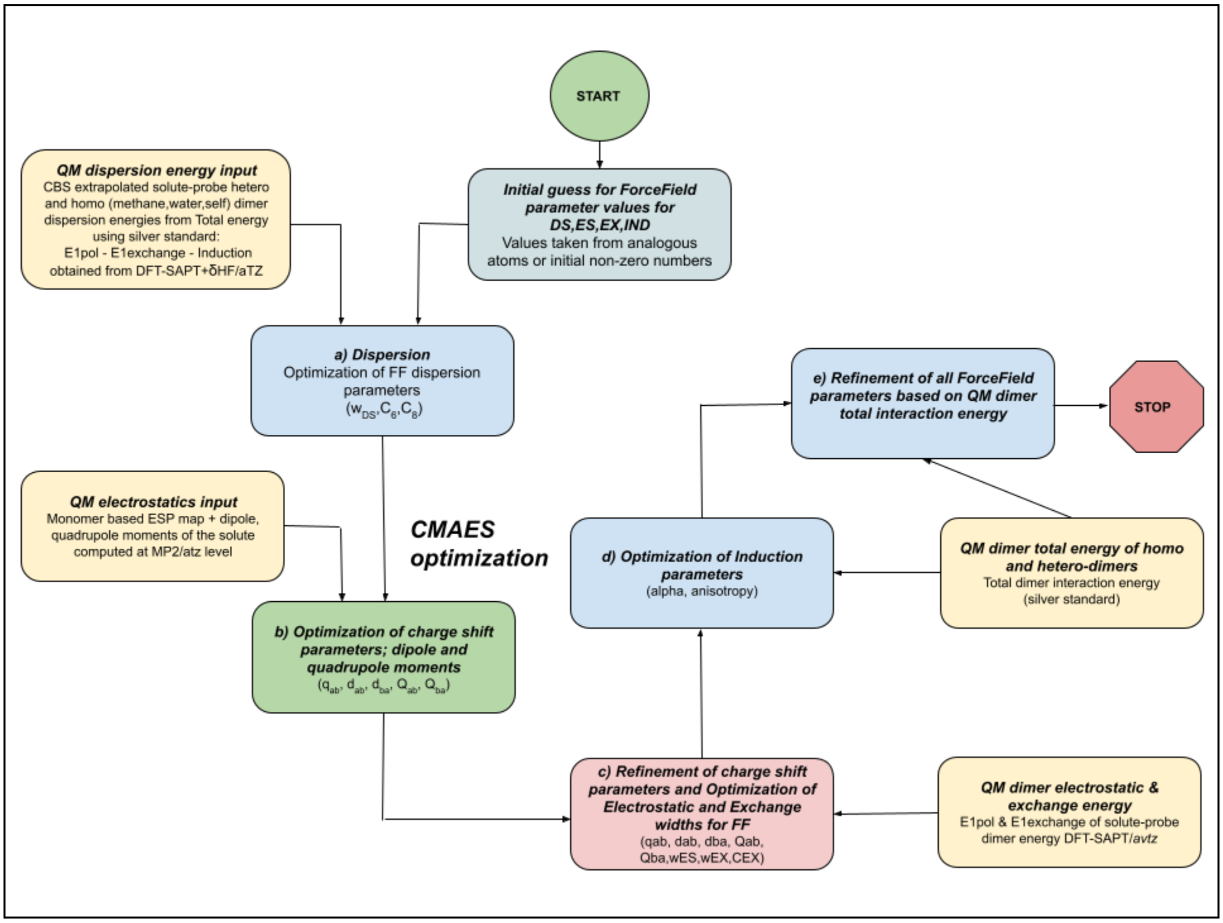

Supplementary Figure 1: Parametrization workflow. This flowchart describes the parametrization processes for obtaining the ARROW FF parameters from QM data.

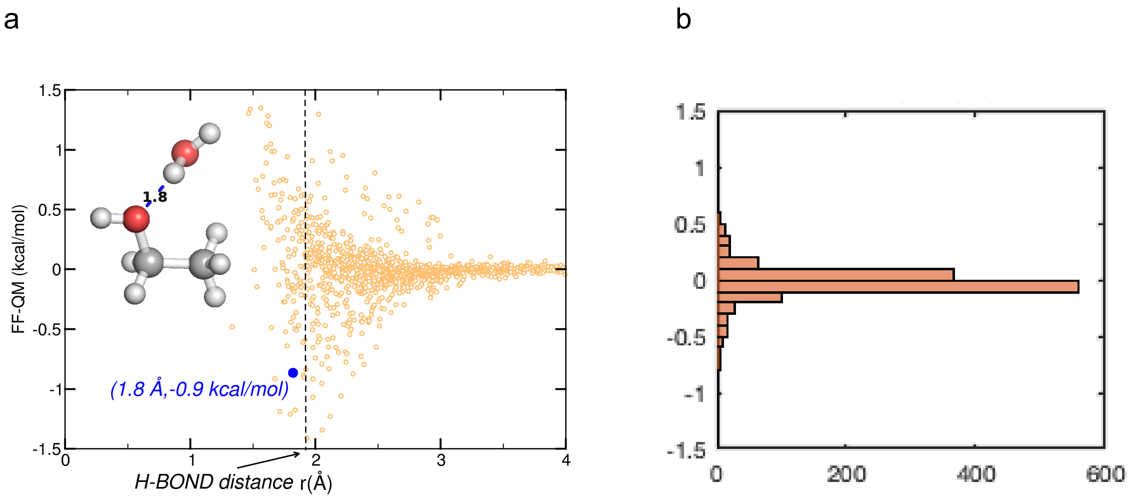

Supplementary Figure 2: FF:QM errors for ethanol-water dimers as a function of closest distance. a) The FF:QM faithfulness becomes worse in the region of close approach and high electron overlap where the electron densities and corresponding integrals have large values and derivatives. The worst low-energy dimer (E - -4.5 kcal/mol, ERR = 0.9 kcal/mol) is shown in the figure. This configuration can be better described by going to a higher (L=3,4) level of the atomic multipole expansion and/or by including multiple Slater exponents, but we chose our truncations of L=2 and one exponent for reasons of computational speed and parameter complexity. The current work shows this is a sufficient cutoff for having a good QM:FF agreement for the overall ensemble of possible configurations. b) Histogram of the total dimers energy errors of ethanol-water.

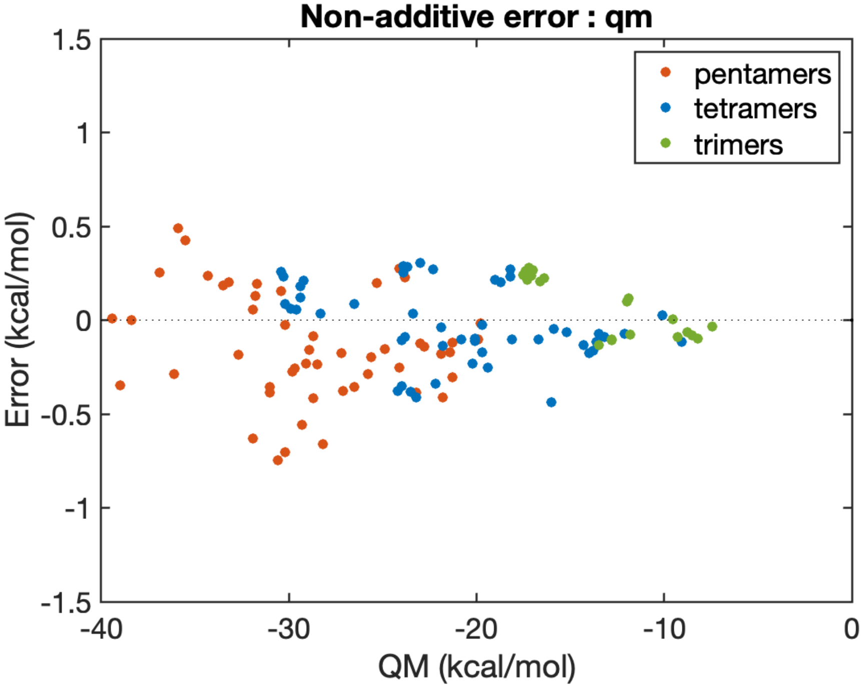

Supplementary Figure 3: **Many-body interactions: specific case ethanol-water multimer.**
The non-additive many-body error for multimers of ethanol with 2,3,4 water molecules correspondingly plotted vs. their total QM intermolecular energy. All the many-body errors are below 1 kcal/mol, or below ~2% of total energy. Multimers of ethanol with 2,3,and 4 water molecules were extracted from molecular dynamics simulations with the ARROW force field. Geometries of those multimers were optimized with the mp2/cc-pVTZ method. Nonadditive energies are full nonbonded energy of MP2/CBS (aug-cc-pVTZ->aug-cc-pVQZ extrapolated) minus energies of dimers nonbonded energy.

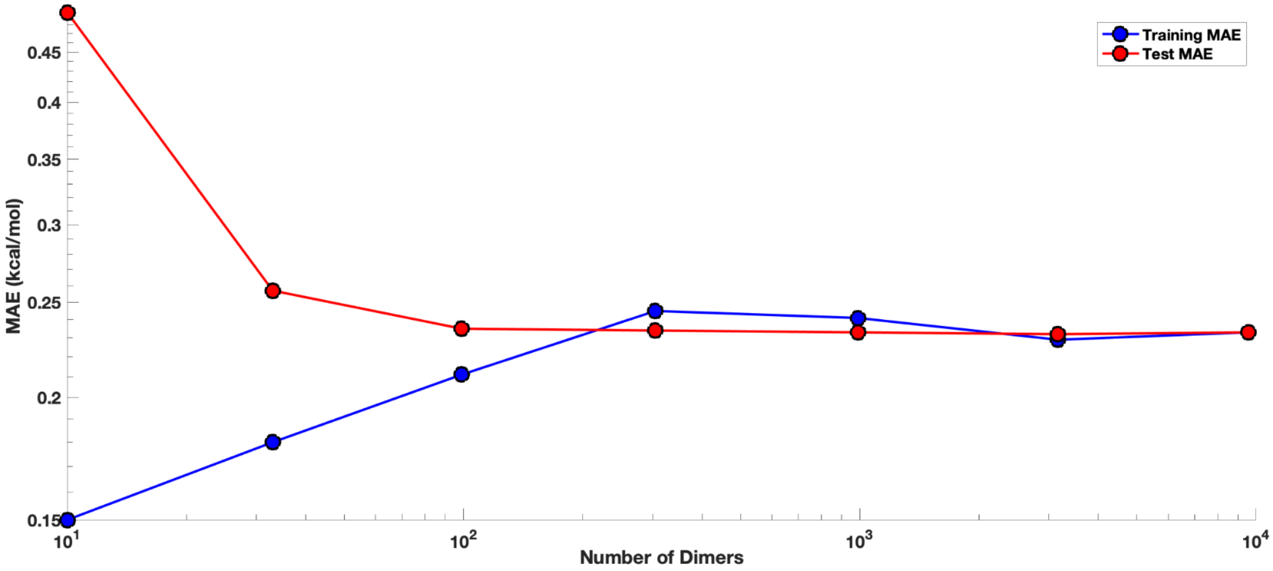

Supplementary Figure 4: **Double logarithmic Force field training-learning curves: n-methyl-acetamide, acetamide and water.** Double logarithm learning plot that was constructed with a subset of the total set of our dimers used in the work. This subset consists of amides (N-methyl-amides, acetamide) and water (9,550 dimers). We use randomly selected configurations from this subset in increasing numbers to construct the plot. This was accomplished using a fixed amount of monomer properties *i. e.* the electrostatic potential surfaces, polarizability tensor etc were kept constant. It is clear that the force field optimization converges almost to the point of saturation around 330 dimers for this subset. As expected for 'formula'- based functional forms, the predictability of the model saturates at its maximum achievable accuracy. In contrast, ML models can theoretically achieve perfect accuracy, but the training data requirements are linear in log-log (accuracy vs training size) space.

a b

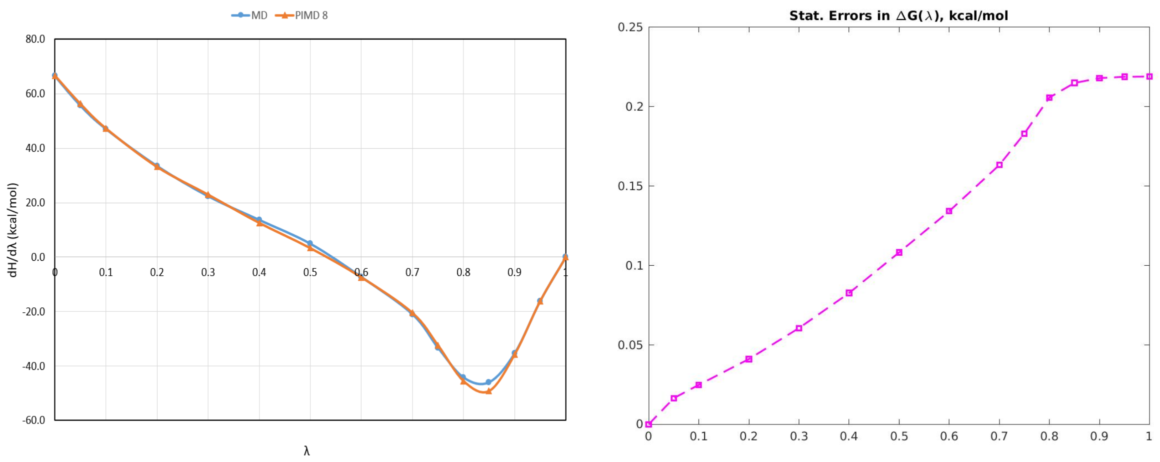

Supplementary Figure 5: The derivative of system Hamiltonian with respect to the alchemical reaction coordinate <dH/d𝜆> for desolvation and its associated statistical errors. a) **<**dH/d𝜆> dependence for desolvation of Anthracene in water. b) Accumulated statistical errors for TI calculations of free energy (ΔG) desolvation of Anthracene in water.

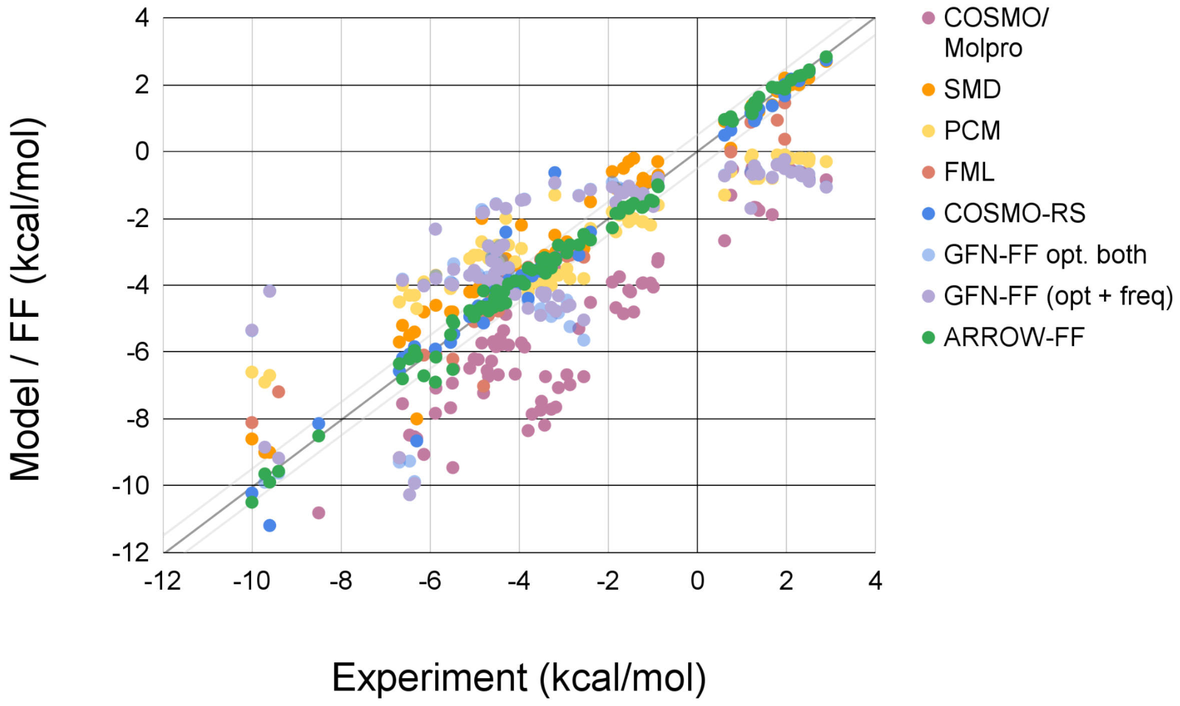

Supplementary Figure 6 Comparisons of free energies of hydration as predicted by implicit solvent models, FML, and ARROW FF. Free energies of hydration of the various organic compounds as predicted by the ARROW FF, *ab initio* derived force field GFN-FF, implicit solvent models and machine learning models.

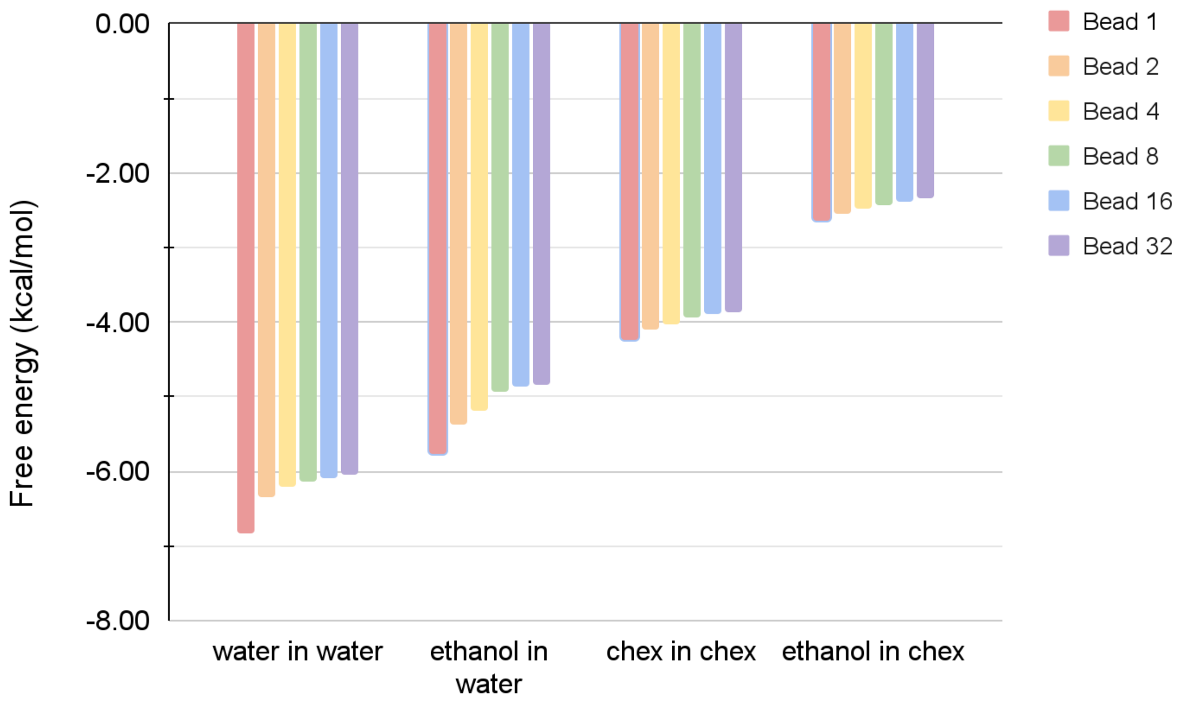

Supplementary Figure 7 PIMD bead convergence on free energy of solvation results. Free energies of hydration and solvation for water, ethanol, and cyclohexane as a function of PIMD bead sizes (1,2,4,8,16,and 32). We see a convergence of results for hydration at bead 8. Above beads 8, the free energy results using BAR are within statistical error of 0.2 kcal/mol.

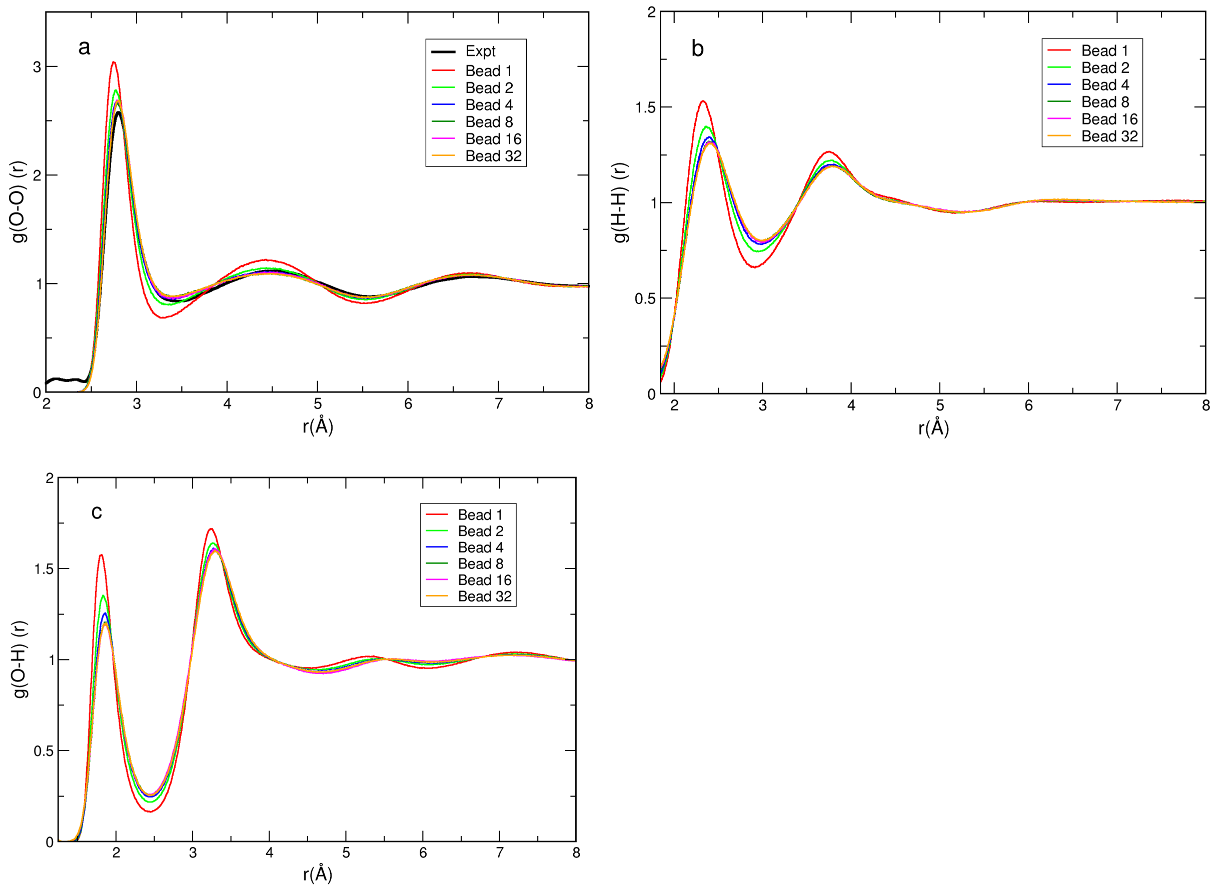

Supplementary Figure 8 PIMD bead convergence for the pair-wise radial distribution functions of water. Pair-wise radial distribution functions (a) O-O, (b) H-H, and (c) H-H as a function of PIMD beads for water at 298 K and 1 bar. Beyond bead 8, we observe a convergence in the magnitude for the various peaks and troughs of the computed pair-distribution functions. The experimental oxygen-oxygen radial distribution function is taken from Skinner et al. 2013. For the O-H and H-H RDF's, no experimental data was reported by Skinner et al.

4. Supplementary References

1. Burns, L. A., Marshall, M. S. & Sherrill, C. D. Appointing silver and bronze standards for noncovalent interactions: a comparison of spin-component-scaled (SCS), explicitly correlated (F12), and specialized wavefunction approaches. *J. Chem. Phys.* **141**, 234111 (2014).
2. Halkier, A., Helgaker, T., Jørgensen, P., Klopper, W. & Olsen, J. Basis-set convergence of the energy in molecular Hartree–Fock calculations. *Chem. Phys. Lett.* **302**, 437–446 (1999).
3. Williams, H. L. & Chabalowski, C. F. Using Kohn−Sham orbitals in symmetry-adapted perturbation theory to investigate intermolecular interactions. *J. Phys. Chem. A* **105**, 646–659 (2001).
4. Misquitta, A. J. & Szalewicz, K. Intermolecular forces from asymptotically corrected density functional description of monomers. *Chem. Phys. Lett.* **357**, 301–306 (2002).
5. Misquitta, A. J., Podeszwa, R., Jeziorski, B. & Szalewicz, K. Intermolecular potentials based on symmetry-adapted perturbation theory with dispersion energies from time-dependent density-functional calculations. *J. Chem. Phys.* **123**, 214103 (2005).
6. Tersoff, J. New empirical approach for the structure and energy of covalent systems. *Phys. Rev. B Condens. Matter* **37**, 6991–7000 (1988).
7. van Duin, A. C. T., Dasgupta, S., Lorant, F. & Goddard, W. A. ReaxFF: A Reactive Force Field for Hydrocarbons. *J. Phys. Chem. A* **105**, 9396–9409 (2001).
8. Grimme, S. A General Quantum Mechanically Derived Force Field (QMDFF) for Molecules and Condensed Phase Simulations. *J. Chem. Theory Comput.* **10**, 4497–4514 (2014).
9. Xu, P., Guidez, E. B., Bertoni, C. & Gordon, M. S. Perspective: Ab initio force field methods derived from quantum mechanics. *J. Chem. Phys.* **148**, 090901 (2018).
10. Greff da Silveira, L., Jacobs, M., Prampolini, G., Livotto, P. R. & Cacelli, I. Development and Validation of Quantum Mechanically Derived Force-Fields: Thermodynamic, Structural, and

Vibrational Properties of Aromatic Heterocycles. *J. Chem. Theory Comput.* **14**, 4884–4900 (2018).

11. Spicher, S. & Grimme, S. Robust Atomistic Modeling of Materials, Organometallic, and Biochemical Systems. *Angew. Chem. Int. Ed Engl.* **59**, 15665–15673 (2020).
12. Donchev, A. G., Galkin, N. G., Pereyaslavets, L. B. & Tarasov, V. I. Quantum mechanical polarizable force field (QMPFF3): refinement and validation of the dispersion interaction for aromatic carbon. *J. Chem. Phys.* **125**, 244107 (2006).
13. Donchev, A. G. et al. Assessment of performance of the general purpose polarizable force field QMPFF3 in condensed phase. *J. Comput. Chem.* **29**, 1242–1249 (2008).
14. Tang, K. T. & Peter Toennies, J. An improved simple model for the van der Waals potential based on universal damping functions for the dispersion coefficients. *The Journal of Chemical Physics* vol. 80 3726–3741 (1984).
15. Axilrod, B. M. & Teller, E. Interaction of the van der Waals Type Between Three Atoms. *J. Chem. Phys.* **11**, 299–300 (1943).
16. Khoruzhii, O. et al. Polarizable Force Fields for Proteins. in *Protein Modelling* (ed. Náray-Szabó, G.) 91–134 (Springer International Publishing, 2014).
17. Jurečka, P., Šponer, J., Černý, J. & Hobza, P. Benchmark database of accurate (MP2 and CCSD(T) complete basis set limit) interaction energies of small model complexes, DNA base pairs, and amino acid pairs. *Phys. Chem. Chem. Phys.* vol. 8 1985–1993 (2006).
18. Rezáč, J., Riley, K. E. & Hobza, P. S66: A well-balanced database of benchmark interaction energies relevant to biomolecular structures. *J. Chem. Theory Comput.* **7**, 2427–2438 (2011).
19. Giese, T. J., Panteva, M. T., Chen, H. & York, D. M. Multipolar Ewald methods, 1: theory, accuracy, and performance. *J. Chem. Theory Comput.* **11**, 436–450 (2015).
20. Martyna, G. J., Klein, M. L. & Tuckerman, M. Nosé–Hoover chains: The canonical ensemble via continuous dynamics. *J. Chem. Phys.* **97**, 2635–2643 (1992).

21. Martyna, G. J., Tuckerman, M. E., Tobias, D. J. & Klein, M. L. Explicit reversible integrators for extended systems dynamics. *Mol. Phys.* **87**, 1117–1157 (1996).
22. Tuckerman, M., Berne, B. J. & Martyna, G. J. Reversible multiple time scale molecular dynamics. *J. Chem. Phys.* **97**, 1990–2001 (1992).
23. Tuckerman, M. E., Berne, B. J., Martyna, G. J. & Klein, M. L. Efficient molecular dynamics and hybrid Monte Carlo algorithms for path integrals. *J. Chem. Phys.* **99**, 2796–2808 (1993).
24. Martyna, G. J., Hughes, A. & Tuckerman, M. E. Molecular dynamics algorithms for path integrals at constant pressure. *J. Chem. Phys.* **110**, 3275–3290 (1999).
25. Feynman, R. P., Hibbs, A. R. & Styer, D. F. *Quantum Mechanics and Path Integrals*. (Courier Corporation, 2010).
26. Kamath, G. et al. Prediction of cyclohexane-water distribution coefficient for SAMPL5 drug-like compounds with the QMPFF3 and ARROW polarizable force fields. *J. Comput. Aided Mol. Des.* **30**, 977–988 (2016).
27. Pereyaslavets, L. et al. On the importance of accounting for nuclear quantum effects in ab initio calibrated force fields in biological simulations. *Proc. Natl. Acad. Sci. U. S. A.* **115**, 8878–8882 (2018).
28. Bennett, C. H. Efficient estimation of free energy differences from Monte Carlo data. *J. Comput. Phys.* **22**, 245–268 (1976).
29. Straatsma, T. P. & McCammon, J. A. Multiconfiguration thermodynamic integration. *J. Chem. Phys.* **95**, 1175–1188 (1991).
30. Weinreich, J., Browning, N. J. & von Lilienfeld, O. A. Machine learning of free energies in chemical compound space using ensemble representations: Reaching experimental uncertainty for solvation. *J. Chem. Phys.* **154**, 134113 (2021).
31. Voityuk, A. A. & Vyboishchikov, S. F. A simple COSMO-based method for calculation of hydration energies of neutral molecules. *Phys. Chem. Chem. Phys.* **21**, 18706–18713

(2019).

32. Klamt, A., Jonas, V., Bürger, T. & Lohrenz, J. C. W. Refinement and Parametrization of COSMO-RS. *J. Phys. Chem. A* **102**, 5074–5085 (1998).

33. Ehlert, S., Stahn, M., Spicher, S. & Grimme, S. Robust and Efficient Implicit Solvation Model for Fast Semiempirical Methods. *J. Chem. Theory Comput.* **17**, 4250–4261 (2021).

34. Alibakhshi, A. & Hartke, B. Improved prediction of solvation free energies by machine-learning polarizable continuum solvation model. *Nat. Commun.* **12**, 3584 (2021).
