# Supplementary material for: Accurate determination of solvation free energies of neutral organic compounds from first principles
Source: Nat Commun. 2022 Jan 20;13:414. doi: 10.1038/s41467-022-28041-0 (PMC8776904; doi:10.1038/s41467-022-28041-0)
Supplement: Supplementary file 2 — Description of Additional Supplementary Files [file 41467_2022_28041_MOESM2_ESM.pdf]

Description of Additional Supplementary Files

File Name: Supplementary Data 1
Description: Solvation data for various functional groups as predicted by ARROW-FF and other models.
